# Tissue-Specific Genetic Control of Splicing: Implications for the Study of Complex Traits

**DOI:** 10.1371/journal.pbio.1000001

**Published:** 2008-12-23

**Authors:** Erin L Heinzen, Dongliang Ge, Kenneth D Cronin, Jessica M Maia, Kevin V Shianna, Willow N Gabriel, Kathleen A Welsh-Bohmer, Christine M Hulette, Thomas N Denny, David B Goldstein

**Affiliations:** 1 Institute for Genome Sciences & Policy, Center for Human Genome Variation, Duke University, Durham, North Carolina, United States of America; 2 Joseph and Kathleen Bryan Alzheimer's Disease Research Center, Duke University, Durham, North Carolina, United States of America; 3 Human Vaccine Institute, Duke University, Durham, North Carolina, United States of America; Genome Institute of Singapore, Singapore

## Abstract

Numerous genome-wide screens for polymorphisms that influence gene expression have provided key insights into the genetic control of transcription. Despite this work, the relevance of specific polymorphisms to in vivo expression and splicing remains unclear. We carried out the first genome-wide screen, to our knowledge, for SNPs that associate with alternative splicing and gene expression in human primary cells, evaluating 93 autopsy-collected cortical brain tissue samples with no defined neuropsychiatric condition and 80 peripheral blood mononucleated cell samples collected from living healthy donors. We identified 23 high confidence associations with total expression and 80 with alternative splicing as reflected by expression levels of specific exons. Fewer than 50% of the implicated SNPs however show effects in both tissue types, reflecting strong evidence for distinct genetic control of splicing and expression in the two tissue types. The data generated here also suggest the possibility that splicing effects may be responsible for up to 13 out of 84 reported genome-wide significant associations with human traits. These results emphasize the importance of establishing a database of polymorphisms affecting splicing and expression in primary tissue types and suggest that splicing effects may be of more phenotypic significance than overall gene expression changes.

## Author Summary

Although humans have a relatively small complement of genes, the proteins encoded by those genes and their biologic function are far more complex. The increased complexity is achieved in part through processes that create different messages from the same gene sequence (alternative splicing) and that regulate the expression of those messages in a tissue-specific fashion. These processes expand the functional capacity of the human genome, but also can create predisposition to disease when these processes go awry. In this study, we investigated how single nucleotide polymorphisms influence both overall gene expression and alternative splicing in two important cell types (brain and blood) highly relevant to human disease. Extensive and tissue-specific regulation of gene expression and alternative splicing were observed in the two tissue types, and some of these polymorphisms were shown to be connected to other polymorphsims that have been recently implicated in human diseases through genome-wide association studies. Most of these connections appeared to relate to alternative splicing as opposed to overall expression changes, suggesting that changes in splicing patterns may be more consequential for disease than those affecting only expression. These data emphasize the importance of comprehensive studies into genetic regulation of gene expression in all human tissue types in order to help understand how genetic variation influences risk of common diseases.

## Abstract 3

We investigated tissue-specific genetic control of gene expression and alternative splicing in primary human cells, and we describe here the implications for understanding how genetic variation influences human disease.

## Introduction

The release of the HapMap data in 2003 and the availability of immortalized cell lines from HapMap participants initiated a new era of research investigating how SNPs affect how genes are expressed at the mRNA level. In 2005, two landmark publications evaluated how SNPs affect overall transcription in immortalized cell line samples collected from unrelated individuals [1,2]. Since those initial publications, the work has advanced with additional studies using more sophisticated microarrays, larger more diverse sample sets, and with studies of heritability of transcript and exon-level expression [3–6].

The work to date however has been limited in scope, largely focusing on the control of overall gene expression in immortalized cells, which may not be representative of in vivo patterns in specific cellular populations [7]. Only two genome-wide studies have focused on human primary cells [8,9], and most studies have considered only overall expression with no attempt to identify polymorphisms that have their effects primarily on alternative splicing.

Here we extend the previous body of work by studying the genetic control of both exon-level and whole-transcript level variation in expression in two primary cell types, including peripheral blood mononucleated cells (PBMCs) and cortical brain tissue from a set of control individuals, combined with parallel genome-wide genotyping of these samples. The implementation of identical genome-wide screens in two primary tissue types has allowed us to identify polymorphisms with clear effects on both overall expression and splicing, and to show that these effects are often tissue specific. We have also established an easy-to-use database that allows users to assess whether a given polymorphism is associated with any local changes in expression and have shown that these data suggest possible underlying causes of several published disease associations.

## Results/Discussion

**Figure 1:**
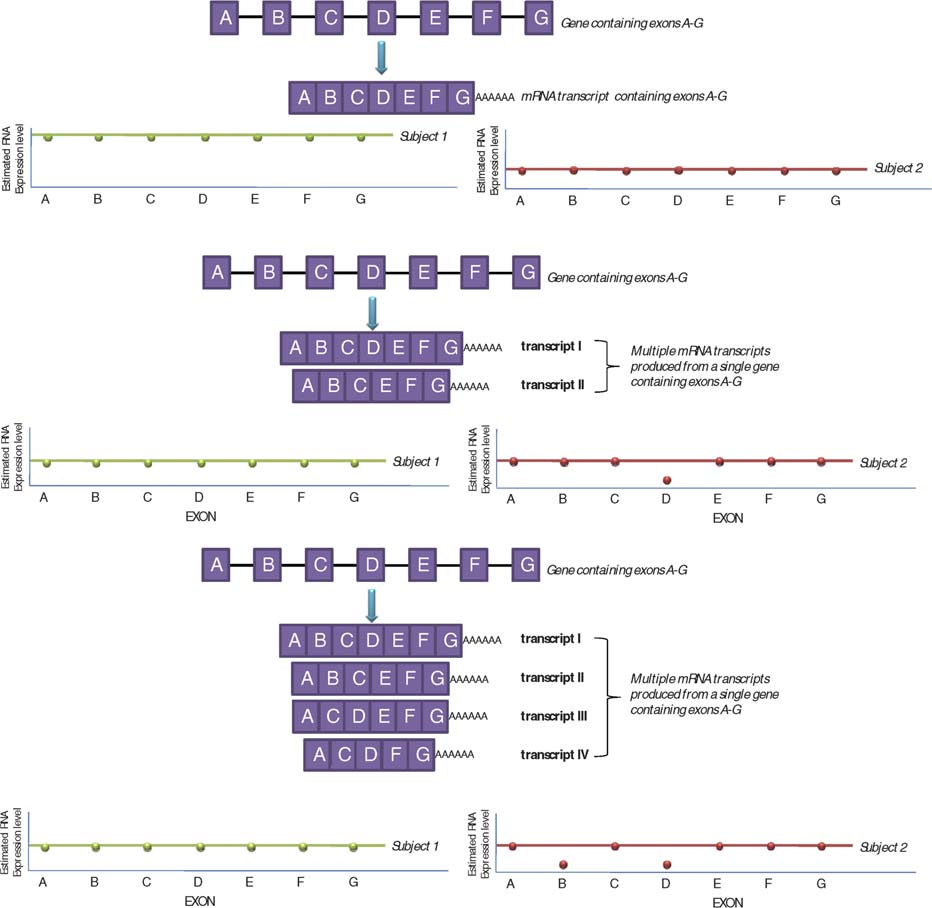
Idealized Representation of How Overall Expression and Alternative Splicing Events Are Reflected in the Exon Array Data Top panel: In this study, all exon-level data were normalized across all exons and individuals. Transcript-level expression was reported for each transcript interrogated on the array by averaging (PLIER method) exon expression levels for all exons contained in a transcript (annotation details can be found at http://www.affymetrix.com). Subject 1 in this example has a higher overall transcript expression level (indicated by green line representing an average of exons A–G within subject 1) compared to subject 2 (red line). In this example, all exons contained in the transcript were expressed at approximately equal levels, suggesting that this transcript does not have alternative splice variants in either subject. Middle panel: An example of the detection of alternative splicing in which multiple transcripts are produced from a single gene through unique combination of the coding regions. Exon D in subject 2 appears to be expressed at lower quantities when compared to the other exons in the transcript (exon D expression levels lie below the average transcript line), indicating that this exon may be spliced out of the transcript in this subject (i.e., higher expression of transcript isoform II). Bottom panel: A scenario where we cannot definitively establish the combinatorial assembly of exons using these data. In this example, subject 2 has lower expression of both exons B and D. We cannot conclude that this subject has a higher proportion of transcript IV expressed compared to subject 1 or if transcript II and III are expressed at higher levels. Despite this shortcoming in situations of large heterogeneity of transcript isoforms produced with multiple alternative splicing events, these data provide a clear indicator of alternative exon composition within transcripts for a given individual. This study was specifically focused on the *cis*-acting genetic regulation of overall expression and splicing. Therefore, we were interested in identifying groups of splicing and expression patterns unique to individuals with the same genotype at certain commonly variant loci in and surrounding the transcript or exon. In cases where the expression of multiple exons was under genetic regulation, we declared it a splicing event if <40% of all exons contained in the transcript were associated with high confidence with the genotype. If >40% of exons were implicated, this was considered to be an overall expression change.

Exon-level microarrays were used to quantify expression levels of fully annotated coding sequences, EST-predicted exons, and bioinformatically predicted exons across the genome. These data allow direct inferences about expression levels of specific exons. By averaging sets of exons it is also possible to estimate expression levels for transcript species (Figure 1, top panel). While the exon-level expression data do not allow inference about the representation of specific (full) transcripts resulting from a given alternative splicing event, they do reflect splicing events in how they influence the proportion of transcripts with and without a given exon (Figure 1, middle and bottom panels).

**Figure 2:**
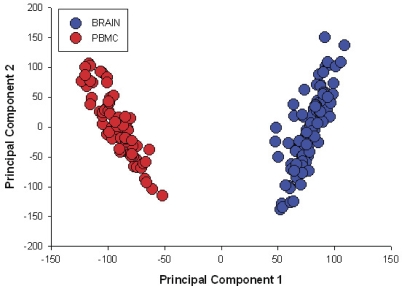
Principal Components Analysis of All Exon Expression Level Data for Both Brain and PBMC Samples The differentiated pattern of expression suggests the need for tissue specific evaluations of alternative splicing and expression and demonstrates the added benefit of studying genetic regulation of splicing and expression in two unique and important cellular populations. A similar profile was observed for the transcript level expression values in the two tissue types suggesting the same level of tissue specificity for splicing.

We first used a principal component analysis to evaluate overall variation in exon-level expression data, and found that tissue source is the most important determinant of that variation (Figure 2). We have therefore implemented genome-wide screens for SNPs controlling gene expression and splicing (referred to as expression quantitative trait locus [eQTL] and splicing quantitative trait locus [sQTL], respectively) separately in the tissue types.

Our screen for (*cis*-acting) polymorphisms controlling expression and splicing evaluated SNPs in or near (within 100 kb) either the target gene or exon. We limited this screen to SNPs with a minor allele frequency (MAF) > 0.04 in our sample sets (requiring at least six alleles to be present in the tissue type investigated). The screen for *cis*-acting sites controlling overall expression and those regulating exon expression levels required approximately ten and 85 million tests, respectively. On average 40 SNPs were considered for each of the ∼22,000 genes, including ∼12 transcripts per gene and ∼four exons per transcript. Thus, thresholds for study-wide significance were 5 × 10^−9^ for transcript level associations and 6 × 10^−10^ for exon-level associations. We identified 584 study-wide significant eQTLs meeting the MAF requirements, but many of these were associated with one another and therefore appeared to reflect the same causal eQTL. We used stepwise regression to eliminate associated SNPs, separately evaluating the two tissue types, and identified 81 independent eQTLs. Significant associations that overlapped between the two tissue types were merged, resulting in 77 transcript level associations. Associations were separated into high confidence (Table 1) and low confidence (Table S2), depending on whether the transcripts were core transcripts, which indicates the highest level of confidence.

**Table 1:**
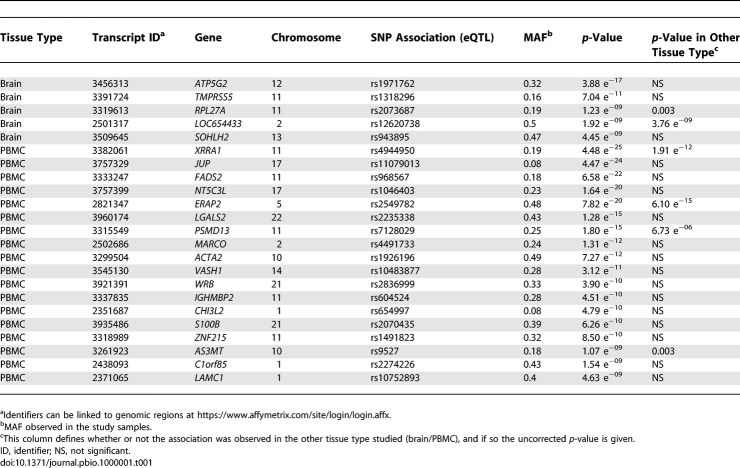
High-Confidence *cis*-Acting SNPs That Were Shown in This Study to Influence Transcript Level Expression

For exon-level assessments 5,357 significant associations were identified in the two tissue types combined and 1,554 remained after removal of associated SNPs. We also removed associations where the probeset contained the associated SNP or a SNP in high linkage disequilibrium (LD, *r* ^2^ > 0.5), leaving 985 associations. Significant associations that overlapped between the two tissue types were merged, resulting in 929 unique exon-level associations. We also identified a subset of these as high confidence (see Table 2 and Table S3) on the basis of the following criteria: (1) *p* < 10^−12^, (2) no reported SNPs within the regions covered by the associated probesets, and (3) no suggested cross-hybridization of the associating probeset.

**Table 2:**
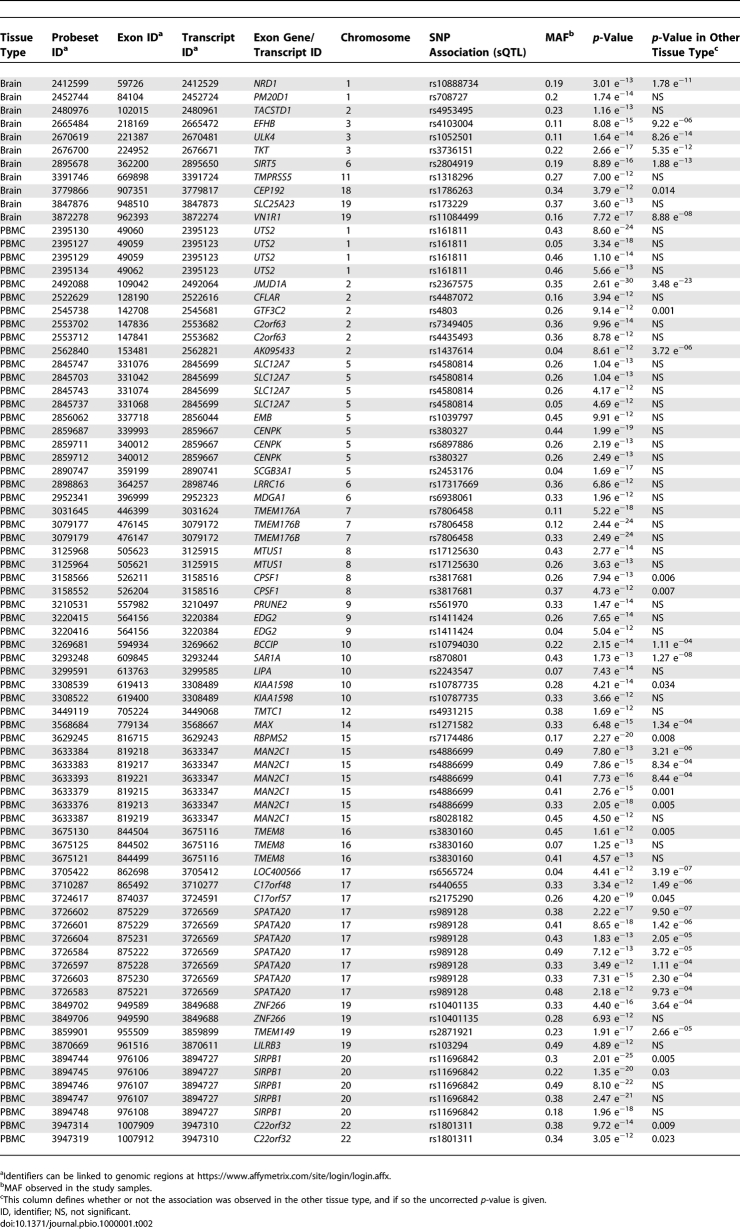
High-Confidence *cis*-Acting SNPs That Were Shown in This Study to Influence Exon-Level Expression

For all high confidence associations identified, we evaluated how often the expression effects of SNPs were observed in the other tissue, and found that 74% of eQTLs and 51% of sQTLs appeared to act exclusively in one tissue or the other. These data clearly indicate a significant role of tissue-specific genetic regulation.

**Figure 3:**
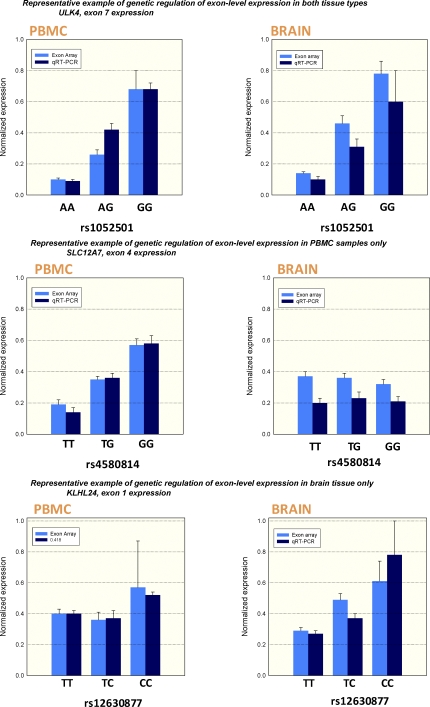
Quantitative Real-Time PCR Confirmation of Selected Genetically Regulated Exon-Level Expression Changes in the Two Tissue Types Three representative scenarios are presented. The top panel shows an sQTL that was present in both brain and PBMCs. The middle panel and bottom panel show sQTLs unique to a particular tissue type, providing unequivocal evidence for tissue specific genetic regulation.

To confirm the accuracy of the exon array technology, and in particular the conclusion of tissue specificity, we selected a subset of sQTLs to evaluate with quantitative real-time PCR (qRT-PCR). Events were selected to replicate the detected event as closely as possible, and also to establish that tissue specificity was not the result of low resolution of the array when exons are differentially expressed in a tissue type. We found a highly significant correlation between measurements using both technologies (Figure 3) with an overall associated *p*-value comparing the two methodologies (linear regression) of 1 × 10^−35^. Importantly, we found clear replication of tissue specificity.

We also evaluated our associations for overlap with previously reported expression QTLs. We confirmed associations with a previously reported eQTL in *LRAP* (renamed *ERAP2*) [1], and also SNP regulation of *RPS26* expression [2,8]. While previous reports document an overall transcript change [2,8,9], we identified the effects of the SNP to be localized to specific exons in the *RPS26* transcript. This discrepancy is probably due to microarray platform differences. We also confirmed in our PBMC sample set several previously reported sQTLs established in HapMap cell lines, including sQTLs in *ULK4, PARP2, C17orf57,* and others [5].

**Figure 4:**
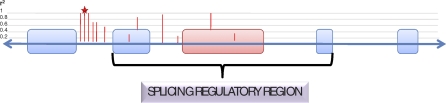
Methodological Details Evaluating the Proximity of a Detected sQTL and Its Region of LD to a Splicing Regulatory Region Red box represents an exon whose expression is correlated with the SNP indicated by the starred red bar. All SNPs in LD with this sQTL are shown by red bars and the height of the bar indicates the level of correlation (*r* ^2^) with the starred SNP. We assessed how often the range of LD for a given sQTL (defined by *r* ^2^ > 0.2 with the sQTL) extended into or surpassed the splicing regulatory region. This analysis was performed by evaluating all mRNA transcripts containing the exon regulated by the sQTL. The splicing regulatory region was defined as the genomic region from the start of the exon located upstream of the associated exon through the stop site of the downstream exon interrogated. If the exon was part of multiple transcripts the region including the most distal and proximal neighboring exons was defined the splicing regulatory region. If the affected exon was located at the beginning or end of the transcript then the range was truncated at the start or stop of the transcript, respectively. Finally, if a single SNP associated with more than one exon in a single transcript they were considered as a single entry in this analysis (i.e., sQTL LD needed only come in close proximity of one of the affected exons to be counted as a positive entry).

Taking the full list of high confidence sQTLs we evaluated how often LD extends into or surpasses regions known to be important in splicing (Figure 4) [10]. We found that 78% of study-wide significant sQTLs, or their extended regions of SNPs in high LD (*r* ^2^ > 0.2), were located near the exons they regulated for at least one transcript containing the exon. The remaining approximately 22% likely reflect unknown exons not screened for in the array, and also possibly novel regulatory regions that regulate splicing outside of these well-documented regions.

Amongst all the study-wide significant sQTLs, only two of them are themselves located in a consensus splicing sequence for the relevant exons, rs10814567 in *POLRE1* and rs7770794 in *PIP3-E*. We note, however, that the probeset screening for the associated exon in *POLRE1* contains a SNP in perfect LD with the consensus site SNP and therefore may be the result of poor hybridization to the target. Given that most common polymorphisms are now known, it is surprising that there are so few cases where a candidate polymorphism responsible for a splicing change is in the consensus sequence, although this scarcity may be due to low primary representation of these SNPs on the array. To further evaluate the role of polymorphisms in consensus sequences, we identified all known polymorphisms in the conserved region located at the exon boundaries of the close to 300,000 core exons measured on the Affymetrix array (three basepairs into the exon and eight into the intron, Ensembl database, National Center for Biotechnology Information [NCBI] Build 36 hg18) and assessed how these influenced the expression levels of neighboring exons. A total of 2,078 SNPs were identified with an MAF > 0.1, of which 1,011 were represented by a proxy on the Illumina genotyping chip (*r* ^2^ = 1 with the splicing SNP in Centre d'Etude du Polymorphisme Humain from Utah (CEU) HapMap samples). For both tissue types, fewer than 7% of consensus site SNPs associated with relevant exon expression levels (Table S4). While it is likely that some associations are missed because of unknown exons not included on the array, this number was surprisingly low given the common conception that disruption of this highly conserved region would very likely disrupt exon assembly. We emphasize that this analysis only evaluates systematically the effects of common SNPs in consensus regions at the exon boundaries and note that rare variation may produce profoundly different effects.

We also assessed transcript-level associations for proximity of LD regions associated with the eQTL to promoter regions (within 10 kb upstream of the transcript start) or in the 3′UTR regions of transcripts, key regions involved in transcription and stability of mRNA transcripts [11]. Twenty-one out of 23 high confidence eQTLs or SNPS in the LD region (*r* ^2^ > 0.2) were found to be located in or extending beyond these relevant regions involved in the steady state expression level of mRNA transcripts.

**Figure 5:**
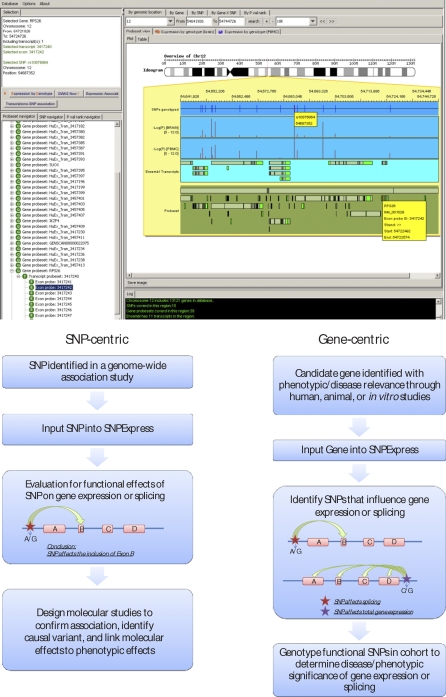
SNPExpress Database Top panel: Output showing an example of an association between rs10876864 and a splicing change in *RPS26*. Software permits the input of an SNP, a gene, or a genomic region for comprehensive interrogation of associations between SNPs and exon/transcript expression levels in the regions surrounding the SNP. The blue frame indicates the SNPs genotyped on the chip, the two lighter blue frames correspond to the −log *p*-values for the brain and PBMC samples, the turquoise panel contains all Ensembl transcripts, and the bottom green panel shows all of the exons/transcripts screened for on the array. Bottom panel: This database can be applied in a SNP-centric or gene-centric approach for determining the functional and phenotypic consequences of genetic variation on the transcriptome.

One motivation for the current project is to facilitate rapid evaluation of whether polymorphisms implicated in human disease influence gene expression or splicing in relevant tissue types. We have therefore established a user interface called SNPExpress, which permits rapid interrogation of the localized effects of common SNPs on exon and transcript level expression (Figure 5). This resource is freely available at: http://people.genome.duke.edu/∼dg48/SNPExpress/.

As of April 2008, >60 genome-wide associations studies were published identifying SNPs with convincing associations to complex human traits. While the association of these SNPs to the study phenotype is secure, how these polymorphisms (or variants associated with them) confer their effects is largely unknown. Of these published genome-wide association scans, 41 papers document genome-wide significant findings for 50 different traits (84 variants). Interestingly, outside of identifying nonsynonymous coding SNPs, only six claim to have identified a functional molecular-level consequence that may contribute to the phenotype, all of which are expression changes at the mRNA transcript level [12–18].

To test the utility of the SNPExpress database, we evaluated the 84 variants (Table S5) for localized associations within the transcript/exon containing this SNP or transcripts/exons within 100 kb of the SNP and determined thirteen to have a strong (*p* < 1 × 10^−5^) effect on an exon or transcript-level expression level (Figure 5, top panel; Table 3). Of these, rs11171739, associated with type 1 diabetes [12], was found through the use of an Illumina proxy to have an association with the exon-level expression of the *RPS26* gene. In a follow-up analysis, a SNP in LD with rs11171739 (rs2292239 *r* ^2^ = 0.71 with rs11171739) was found to be more highly associated with type 1 diabetes [19]. Interestingly, a SNP located upstream of both of these SNPs, rs10876864, was found in our dataset to have the strongest association with a splicing event in *RPS26*. This observation extends what was recently reported by Schadt et al. [9] by specifically identifying *RPS26* splicing as responsible for the expression association with the implicated polymorphism in type 1 diabetes. More generally, however, these results illustrate the exceptional difficulty of moving from phenotypic associations to underlying biological mechanisms. While the strong splicing association with *RPS26* makes it a convincing candidate for being responsible for the diabetes risk, the originally reported polymorphism is located in the *ERBB3* gene, which also has been suggested as having direct relevance in type 1 diabetes [19]. Fortunately, the effects may in this case be resolvable because rs10876864 has a stronger association with the splicing change than does rs2292239. The association between these polymorphisms, while high, is not complete and it should be possible to resolve which SNP is the more likely causal variant by testing whether the originally identified polymorphism has a stronger association with type 1 diabetes than does the polymorphism more strongly associated with the splicing change.

**Table 3:**
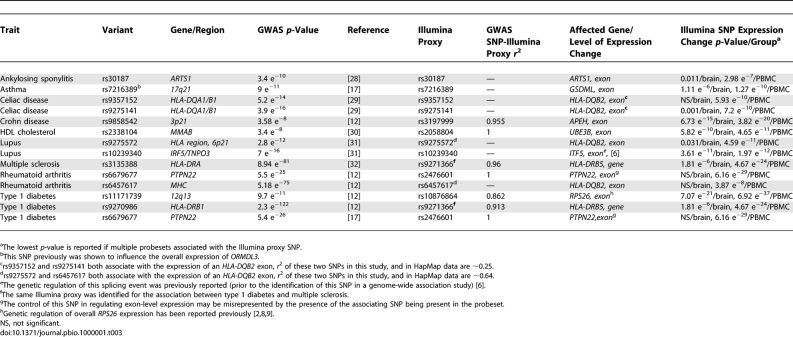
Overlap between e- and sQTLs and Genome-Wide Significant Associations with Complex Human Traits

This scan of genome-wide associations for effects on expression changes also identified splicing effects of rs6678677, a SNP originally identified as a risk factor in rheumatoid arthritis and later a contributor to type 1 diabetes predisposition, in the *PTPN22* gene [12,19–21]. We note that the associated SNP is located directly in the region targeted by the associated probeset, which may result in a false positive association, however the SNP effects were not observed in the brain tissue despite a similar expression level in the minor allele homozygotes. Additional work is needed to confirm this association.

Other e- or sQTLs that overlapped with associations in genome-wide association studies were found for SNPs previously implicated in ankylosing sponylitis, asthma, celiac disease, Crohn disease, HDL cholesterol, lupus, multiple sclerosis rheumatoid arthritis, and type 1 diabetes. It is unclear why the majority of the splicing/expression associations we have found are for SNPs originally implicated in autoimmune diseases. Although this result could reflect a particular importance of splicing variation in autoimmunity, two other possibilities seem more plausible. First, the imbalance could be the result of a methodological bias in evaluating a tissue type clearly relevant to immune system function (PBMCs). While brain tissue was included, little progress has been made identifying common variants that influence brain-specific phenotypes in genome-wide studies. Interestingly, for each e- or sQTL the association in the immune system relevant PBMCs in all cases was stronger compared to that observed in the brain tissue samples (Table 3), which argues for the importance of assessing expression and splicing effects in tissue types most relevant to the disease under study.

The second possible explanation for the clear excess of candidate mechanisms in the case of autoimmune diseases is more fundamental and relates to the growing recognition of the importance of rare variants in common disease [22–24]. It is generally assumed that when a common SNP is associated with disease in a genome-wide study, that it, or some other common variant in LD with it, is responsible for the association. It is theoretically possible, however, that many of the associations observed are not due to single common variants, but rather due to a constellation of more rare disease-causing variants that happen to occur, by chance, more frequently along with one of the common alleles at given SNP as opposed to the other. In such a case, the signal of association credited to a common SNP is actually a synthetic association resulting from the contributions of multiple rare SNPs. In such cases a screen for a common SNP associated with an underlying biological effect (such as expression or splicing) is not likely to identify a causal site. Our failure to identify any good strong candidate SNPs controlling expression or splicing associated with disease implicated SNPs in conditions other than autoimmune conditions could reflect a difference in the importance of common variants in autoimmune disease versus other diseases. Such a difference in the role of common variants could be an indirect consequence of selection [25] related to infectious disease, which has created predispositions to autoimmune conditions. In short, outside of autoimmunity, it is possible that many of the reported associations are synthetic, due to multiple rare variants, and therefore the reason that no clear expression or splicing effects have been consistently identified at these loci.

A key challenge in human disease genomics is establishing appropriate resources to elucidate the underlying biological causes of polymorphisms that are associated with disease. As demonstrated here, one key element in this effort is the development of appropriate databases that describe the relationship between polymorphisms and patterns of gene expression and splicing in multiple human primary tissue types. As the field transitions to the study of rare variants it will be critical to supplement these datasets with complete DNA resequencing data to comprehensively characterize the full spectrum of genetic regulation of expression.

## Methods

### Samples.

Brain tissue samples (frontal cortex) from neurologically healthy control individuals were obtained from the National NeuroAIDS Tissue Consortium (NNTC), the Kathleen Price Bryan Brain Bank (KPBBB) at Duke University, and the Oregon Brain Bank. PBMCs from healthy living participants were purchased from Seracare Bioservices, Cellular Technology Ltd., and also provided by the Duke Human Vaccine Institute. All samples used in this analysis were of European ancestry. Sample demographics are included in Table S1. PBMCs obtained from living participants were completely de-identified and obtained according to standards set forth by the Duke University Institutional Review Board.

### Genome-wide expression and genotyping.

Affymetrix Human ST 1.0 exon arrays were used to assess exon and transcript expression levels for all samples used in the study. Genome-wide genotyping was performed using Illumina Human Hap550K chips. DNA and RNA were extracted using standard Qiagen protocols. Exon array sample preparation from total RNA was conducted based on standard Affymetrix protocols.

Exon array data were evaluated using a series of quality control steps defined by Affymetrix for uniform hybridization intensity, abnormal background signals, and sample outliers. The data were normalized across all samples for a tissue type on an exon and transcript level (four separate normalizations) per Affymetrix PLIER protocol with a sketch-quantile normalization procedure (Affymetrix Expression Console). This algorithm also removed undetectable signals for the dataset using a screen for signals below a group of antigenomic probesets. Principal component analysis (PCA) was performed to look secondarily to identify sample outliers using Partek Genomics Suite. Individual sample positions on top principal component (PC) axes were exported and the effects postmortem interval (applicable only to brain tissue analyses), age, gender, and sample source/processing day were tested for significance using STATA/IC 10.0. All of the four covariates were deemed to impact the sources of variability on both an exon and transcript level, and were therefore included in subsequent genetic association analyses as covariates in linear regression models. A combined normalization on both brain tissue samples and PBMCs also was performed on both the exon and transcript level. Principal components analyses were performed for a combined normalization in order to demonstrate the unique expression patterns in cortical tissue and PBMCs.

Genotyping quality was assessed using previously published methods [13]. Briefly, all SNPs that we called with a genotyping frequency of >99% across individuals (1% rule) were included in the analysis. All participants were also required to have a genotyping success rate of >99% for all SNPs that passed the 1% rule. Finally, each study-wide significant SNP identified in this analysis was manually evaluated in the Illumina Bead Studio files for genotyping quality/accuracy.

### Quantitative real-time PCR.

Taqman-based real-time PCR was used to confirm exon-level expression changes. Primers and fluorescently-labeled probes were custom designed for specific detection of exon-level expression. The follow primers/probes were used: ULK4, TCTCGTCCTAAAGCTTCTTCAGATT; ULK4, CTTTTCTGAGGATCTCTTTGAAGT; ULK4.PROBE, VIC- ATTAATTTGCTTGATGGGTT; SLC12A7F, ATCCTGGGCGTCATCCTCT; SLC12A7R, CACATGGCCACGATGAGG; SLC12A7.PROBE, VIC-CTGGTGTCCTGGAGTCCT; KLHL24F, TGGTACTAATATTGGGACGCAGAC; KLHL24R, CGCTTAGTTGCTGGGGAATC; KLHL24.PROBE, VIC- TAAACAGAGAGGATCTTGGG.

FAM-labeled β-actin was used as an internal control in a multiplex reaction. Assays were performed according to standard methods (900-nM primer and 250-nM probe in 20-μl reaction mix, Applied Biosystems). Fluorescence outputs were quantified in real time using a 7900HT Fast Real Time PCR System and the data were analyzed using SDS software v.2.2.2 (Applied Biosystems).

### Statistical analyses.

To screen for *cis*-acting genetic regulation of splicing/expression genetic association analyses were conducted to search for *cis*-acting SNPs that regulate exon-level and gene-level expression. Specifically, associations were limited to SNPs lying in or 100 kb surrounding the region of the transcripts or exons. Linear regression incorporating all the covariates was performed using PLINK genome-wide association analysis toolkit (http://pngu.mgh.harvard.edu/∼purcell/plink/) [26]. To control for the possibility of spurious associations resulting from population stratification, we used a modified EIGENSTRAT method [13,27]. A total of four separate analyses were conducted, including PBMC transcript level, PBMC exon level, brain transcript level, and brain exon level. Thresholds for significance at each level were calculated based on the total number of association tests conducted within the four separate analyses.

Initially, all SNPs were excluded from analysis if the minor allele was not present at least six times in the sample group, translating to an MAF cutoff of 0.04. All significant observations that had *p*-values below the threshold and that met the MAF cutoff requirement were exported and the list was evaluated based on the following criteria: (1) Associations that were present on the exon or transcript list that were actually due to the opposing level were moved to the appropriate list. Specifically, a study-wide significant transcript level association was reported if the *p*-value achieved the threshold requirements, the affected transcript contained >2 exons, and >40% of exons contained in a transcript were significantly associated at exon-level study-wide significance level. Consistent with those rules, transcripts containing <2 exons associating with genotype that were study-wide significant and/or <40% of exons within a transcript were affected were removed and allowed on the exon-level list if they achieved exon-level study-wide significance. (2) Using stepwise linear regression (STATA/IC 10.0) associations were removed that were redundant due to LD between SNPs. Specifically, following inclusion of the most significant SNP-probeset association, only SNPs that contributed significantly above and beyond the initial association at a *p*-value of <10^−6^ were considered as a separate association. (3) Finally, if the sQTL or a SNP in LD (*r* ^2^ > 0.5) was located in a probeset for the exon-level associations, it was excluded from any list of significant associations. Step 3 was not applied to transcript level associations as these expression levels were determined over a range of exons thereby reducing the contribution of SNPs to the expression levels. Post hoc evaluation of the transcript level associations we identified were manually inspected for effects of SNPs or SNPs in LD contributing to the association by exporting the raw values, eliminating probesets that contained a SNP, and recalculating the expression level. None of the reported associations could be accounted for by a SNP in a probeset measuring the transcript.

We also assessed whether associations were present in the other sample type. The *p*-value for declaring an effect in the other tissue was based on a *p*-value cutoff of 0.05. Directionality was confirmed to be the same in the two tissue types for all overlapping associations. All uncorrected *p*-values for observations in the other tissue types are provided in the relevant tables.

See Figure 4 for methodological details regarding the screen for proximity of sQTLs to affected exons.

To screen for effects of consensus site SNPs, we first identified all known SNPs located in highly conserved consensus site regions at the exon-intron boundary including all SNPs eight basepairs into an intron and three basepairs into an exon (Ensembl database). A total of 2,078 common consensus site SNPs were identified, of which 1,011 had proxies (*r* ^2^ = 1) with one or more SNPs on the Illumina Human Hap550K chip allowing assessments of their effects in the present dataset. Specifically, consensus site SNPs, or their proxies, were assessed for significant associations (uncorrected *p* < 0.05) with the expression level of the immediate exon or exons located up- or downstream for all transcripts containing that exon.

## Supporting Information

Table S1 Sample Demographics (48 KB RTF)

Table S2 Lower Confidence *cis*-Acting SNPs That Were Shown in This Study to Influence Transcript Level Expression (321 KB RTF)

Table S3 Lower Confidence *cis*-Acting SNPs That Were Shown in This Study to Influence Exon-Level Expression (4.3 MB RTF)

Table S4 Table of Associations between Consensus Site SNPs and Adjacent Exons (285 KB RTF)

Table S5 A List of Genome-wide Association Studies Interrogated for Significant Associations That Affect Expression and Splicing in the SNPExpress Database. Full references are provided below this table.
(459 KB RTF)

## Abbreviations

eQTL: expression quantitative trait locus
LD: linkage disequilibrium
MAF: minor allele frequency
PBMC: peripheral blood mononucleated cell
sQTL: splicing quantitative trait locus

## References

[pbio-1000001-b001] Cheung VG, Spielman RS, Ewens KG, Weber TM, Morley M (2005). Mapping determinants of human gene expression by regional and genome-wide association. Nature.

[pbio-1000001-b002] Stranger BE, Forrest MS, Clark AG, Minichiello MJ, Deutsch S (2005). Genome-wide associations of gene expression variation in humans. PLoS Genet.

[pbio-1000001-b003] Dixon AL, Liang L, Moffatt MF, Chen W, Heath S (2007). A genome-wide association study of global gene expression. Nat Genet.

[pbio-1000001-b004] Hull J, Campino S, Rowlands K, Chan MS, Copley RR (2007). Identification of common genetic variation that modulates alternative splicing. PLoS Genet.

[pbio-1000001-b005] Kwan T, Benovoy D, Dias C, Gurd S, Provencher C (2008). Genome-wide analysis of transcript isoform variation in humans. Nat Genet.

[pbio-1000001-b006] Zhang W, Duan S, Kistner EO, Bleibel WK, Huang RS (2008). Evaluation of genetic variation contributing to differences in gene expression between populations. Am J Hum Genet.

[pbio-1000001-b007] Yanai I, Benjamin H, Shmoish M, Chalifa-Caspi V, Shklar M (2005). Genome-wide midrange transcription profiles reveal expression level relationships in human tissue specification. Bioinformatics.

[pbio-1000001-b008] Myers AJ, Gibbs JR, Webster JA, Rohrer K, Zhao A (2007). A survey of genetic human cortical gene expression. Nat Genet.

[pbio-1000001-b009] Schadt EE, Molony C, Chudin E, Hao K, Yang X (2008). Mapping the genetic architecture of gene expression in human liver. PLoS Biol.

[pbio-1000001-b010] Cartegni L, Chew SL, Krainer AR (2002). Listening to silence and understanding nonsense: exonic mutations that affect splicing. Nat Rev Genet.

[pbio-1000001-b011] Shyu AB, Wilkinson MF, van Hoof A (2008). Messenger RNA regulation: to translate or to degrade. Embo J.

[pbio-1000001-b012] Wellcome Trust Case Control Consortium (2007). Genome-wide association study of 14,000 cases of seven common diseases and 3,000 shared controls. Nature.

[pbio-1000001-b013] Fellay J, Shianna KV, Ge D, Colombo S, Ledergerber B (2007). A whole-genome association study of major determinants for host control of HIV-1. Science.

[pbio-1000001-b014] Hom G, Graham RR, Modrek B, Taylor KE, Ortmann W (2008). Association of systemic lupus erythematosus with C8orf13-BLK and ITGAM-ITGAX. N Engl J Med.

[pbio-1000001-b015] Kathiresan S, Melander O, Guiducci C, Surti A, Burtt NP (2008). Six new loci associated with blood low-density lipoprotein cholesterol, high-density lipoprotein cholesterol or triglycerides in humans. Nat Genet.

[pbio-1000001-b016] Libioulle C, Louis E, Hansoul S, Sandor C, Farnir F (2007). Novel Crohn disease locus identified by genome-wide association maps to a gene desert on 5p13.1 and modulates expression of PTGER4. PLoS Genet.

[pbio-1000001-b017] Moffatt MF, Kabesch M, Liang L, Dixon AL, Strachan D (2007). Genetic variants regulating ORMDL3 expression contribute to the risk of childhood asthma. Nature.

[pbio-1000001-b018] Thorleifsson G, Magnusson KP, Sulem P, Walters GB, Gudbjartsson DF (2007). Common sequence variants in the LOXL1 gene confer susceptibility to exfoliation glaucoma. Science.

[pbio-1000001-b019] Todd JA, Walker NM, Cooper JD, Smyth DJ, Downes K (2007). Robust associations of four new chromosome regions from genome-wide analyses of type 1 diabetes. Nat Genet.

[pbio-1000001-b020] Plenge RM, Padyukov L, Remmers EF, Purcell S, Lee AT (2005). Replication of putative candidate-gene associations with rheumatoid arthritis in >4,000 samples from North America and Sweden: association of susceptibility with PTPN22, CTLA4, and PADI4. Am J Hum Genet.

[pbio-1000001-b021] Bottini N, Musumeci L, Alonso A, Rahmouni S, Nika K (2004). A functional variant of lymphoid tyrosine phosphatase is associated with type I diabetes. Nat Genet.

[pbio-1000001-b022] Stefansson H, Rujescu D, Cichon S, Pietilainen OP, Ingason A (2008). Large recurrent microdeletions associated with schizophrenia. Nature.

[pbio-1000001-b023] Walsh T, McClellan JM, McCarthy SE, Addington AM, Pierce SB (2008). Rare structural variants disrupt multiple genes in neurodevelopmental pathways in schizophrenia. Science.

[pbio-1000001-b024] Weiss LA, Shen Y, Korn JM, Arking DE, Miller DT (2008). Association between microdeletion and microduplication at 16p11.2 and autism. N Engl J Med.

[pbio-1000001-b025] Gibson G, Goldstein DB (2007). Human genetics: the hidden text of genome-wide associations. Curr Biol.

[pbio-1000001-b026] Purcell S, Neale B, Todd-Brown K, Thomas L, Ferreira MA (2007). PLINK: a tool set for whole-genome association and population-based linkage analyses. Am J Hum Genet.

[pbio-1000001-b027] Price AL, Patterson NJ, Plenge RM, Weinblatt ME, Shadick NA (2006). Principal components analysis corrects for stratification in genome-wide association studies. Nat Genet.

[pbio-1000001-b028] Burton PR, Clayton DG, Cardon LR, Craddock N, Deloukas P (2007). Association scan of 14,500 nonsynonymous SNPs in four diseases identifies autoimmunity variants. Nat Genet.

[pbio-1000001-b029] van Heel DA, Franke L, Hunt KA, Gwilliam R, Zhernakova A (2007). A genome-wide association study for celiac disease identifies risk variants in the region harboring IL2 and IL21. Nat Genet.

[pbio-1000001-b030] Willer CJ, Sanna S, Jackson AU, Scuteri A, Bonnycastle LL (2008). Newly identified loci that influence lipid concentrations and risk of coronary artery disease. Nat Genet.

[pbio-1000001-b031] Harley JB, Alarcon-Riquelme ME, Criswell LA, Jacob CO, Kimberly RP (2008). Genome-wide association scan in women with systemic lupus erythematosus identifies susceptibility variants in ITGAM, PXK, KIAA1542 and other loci. Nat Genet.

[pbio-1000001-b032] Hafler DA, Compston A, Sawcer S, Lander ES, Daly MJ (2007). Risk alleles for multiple sclerosis identified by a genomewide study. N Engl J Med.

