# Supplementary material for: Tissue-Specific Genetic Control of Splicing: Implications for the Study of Complex Traits
Source: PLoS Biol. 2008 Dec 23;6(12):e1000001. doi: 10.1371/journal.pbio.1000001 (PMC2605930; doi:10.1371/journal.pbio.1000001)
Supplement: Table S1 — (48 KB RTF) [file pbio.1000001.st001.rtf]

Table S1.  Sample demographics.  		
				
Sample type	Age [mean yrs (min-max)]	Gender	Postmortem intervala  [mean hrs (min-max)]	
BRAIN (n=93)	74 (34 - >90)	males: 55	14 (1.2 - 46)	
		females: 38		
PBMC (n=80)	39 (18 - 67)	males: 52	---	
		females: 28		
aThe interval between time of death and time of brain tissue procurement 	
