# Supplementary material for: Tissue-Specific Genetic Control of Splicing: Implications for the Study of Complex Traits
Source: PLoS Biol. 2008 Dec 23;6(12):e1000001. doi: 10.1371/journal.pbio.1000001 (PMC2605930; doi:10.1371/journal.pbio.1000001)
Supplement: Table S2 — (321 KB RTF) [file pbio.1000001.st002.rtf]

Table S2.   Lower confidence cis-acting SNPs that were shown in this study to influence transcript level expression.  
				
TISSUE TYPE	Transcript IDc	LEVELa	Gene	Chr	SNP assoc (eQTL)	MAFd	p value	p value in other tissue typeb					
BRAIN	3354666	full	GENSCAN00000012571	11	rs11219981	0.226	2.22E-25	NS					
BRAIN	3562320	full	GENSCAN00000003902	14	rs17181608	0.188	1.67E-21	NS					
BRAIN	2798178	extended	-	4	rs4863140	0.048	5.97E-21	9.23E-16					
BRAIN	3404222	extended	OVOS2	12	rs7310367	0.366	2.95E-19	NS					
BRAIN	2798176	full	GENSCAN00000013980	4	rs4863140	0.048	3.11E-19	1.19E-12					
BRAIN	3058560	full	-	7	rs4143262	0.306	8.15E-19	NS					
BRAIN	2376680	extended	-	1	rs708727	0.43	1.18E-16	0.01					
BRAIN	3053435	extended	BC044608	7	rs4718180	0.403	1.92E-15	2.29E-09					
BRAIN	3533878	full	-	14	rs10149831	0.409	3.55E-15	NS					
BRAIN	3372403	extended	-	11	rs10838738	0.349	7.38E-15	5.54E-12					
BRAIN	3831806	extended	BC041478	19	rs320887	0.328	1.11E-13	NS					
BRAIN	3802866	full	-	18	rs16947857	0.043	1.31E-13	NS					
BRAIN	3723866	extended	-	17	rs2532269	0.211	2.65E-13	1.15E-15					
BRAIN	3444528	extended	-	12	rs6488331	0.188	4.80E-13	9.98E-15					
BRAIN	2798157	extended	-	4	rs4863140	0.048	5.27E-13	0.001					
BRAIN	3710292	extended	-	17	rs440655	0.478	6.47E-13	8.81E-07					
BRAIN	3760247	extended	-	17	rs2668692	0.213	2.71E-12	2.34E-05					
BRAIN	2843905	extended	-	5	rs4700752	0.403	3.17E-12	NS					
BRAIN	3793986	extended	-	18	rs522009	0.253	3.43E-12	NS					
BRAIN	3159862	full	-	9	rs7868180	0.065	6.19E-12	NS					
BRAIN	3723864	extended	-	17	rs2532269	0.211	6.65E-12	1.07E-09					
BRAIN	3140398	extended	-	8	rs16938215	0.048	1.16E-11	NS					
BRAIN	3461492	extended	-	12	rs698132	0.366	1.36E-11	3.88E-04					
BRAIN	2481469	full	-	2	rs10205982	0.043	1.27E-10	NS					
BRAIN	3503376	extended	MRPL3	13	rs1806658	0.371	1.34E-10	NS					
BRAIN	3584251	extended	-	15	rs12904856	0.156	1.52E-10	NS					
BRAIN	3055296	full	-	7	rs757839	0.097	1.66E-10	NS					
BRAIN	3905332	extended	SNORA71A	20	rs752774	0.194	1.84E-10	0.013					
BRAIN	3366297	full	GENSCAN00000005600	11	rs7128766	0.075	2.25E-10	NS					
BRAIN	3635184	extended	AK094053	15	rs934136	0.333	2.74E-10	3.84E-13					
BRAIN	2409708	full	GENSCAN00000036602	1	rs1417371	0.059	2.92E-10	NS					
BRAIN	3488727	extended	-	13	rs6314	0.086	3.23E-10	NS					
BRAIN	2359243	extended	LOC339400	1	rs12130219	0.277	3.61E-10	0.014					
BRAIN	3282114	full	GENSCAN00000057438	10	rs7896781	0.364	4.15E-10	9.40E-05					
BRAIN	2527305	extended	-	2	rs2541403	0.478	5.20E-10	NS					
BRAIN	3883672	extended	-	20	rs2050930	0.194	6.13E-10	4.94E-07					
BRAIN	3868654	extended	-	19	rs2560935	0.059	7.54E-10	NS					
BRAIN	3069607	extended	-	7	rs17140937	0.076	8.12E-10	NS					
BRAIN	3519587	full	-	13	rs9546436	0.043	9.55E-10	NS					
BRAIN	3372408	full	-	11	rs10838738	0.349	1.03E-09	1.83E-04					
BRAIN	3915388	extended	AL355741	21	rs12626360	0.398	1.05E-09	NS					
BRAIN	3443924	extended	uc001qwo.1	12	rs10505741	0.065	1.18E-09	0.022					
BRAIN	3500441	extended	-	13	rs3759453	0.398	1.27E-09	NS					
BRAIN	3867728	extended	-	19	rs3810185	0.048	2.12E-09	NS					
BRAIN	2515330	extended	-	2	rs6754817	0.306	3.06E-09	0.001					
BRAIN	3713487	extended	TRIM16L	17	rs3913332	0.204	3.15E-09	NS					
BRAIN	3409175	extended	-	12	rs4931075	0.231	3.62E-09	NS					
PBMC	3187510	extended	LOC253039	9	rs10760117	0.419	3.02E-30	3.30E-13					
PBMC	4047671	extended	HuEx_Tran_4047671	6	rs9271366	0.169	4.67E-24	1.81E-06					
PBMC	3939608	extended	DDTL	22	rs4822458	0.45	4.58E-11	1.35E-07					
PBMC	3589872	extended	C15orf23	15	rs7166991	0.175	1.45E-10	0.003					
PBMC	3856524	extended	uc002nqe.1	19	rs7252706	0.488	3.64E-10	0.001					
PBMC	3641823	extended	PRKXP1	15	rs12460	0.494	3.14E-09	4.17E-04					
PBMC	3707584	extended	AK056005	17	rs1047966	0.144	4.61E-09	0.018					
a	Level is defined by Affymetrix, depending on the level of literature support for the exon, with confidence hierarchy core>extended>full>free.	
b	This column defines whether or not the association was observed in the other tissue type studied (brain/PBMC) in the designated cohort, and if so the uncorrected p value is given.	
c	Identifiers can be linked to genomic regions at  https://www.affymetrix.com/site/login/login.affx	
d	MAF observed in the study sample	
