# Supplementary material for: Tissue-Specific Genetic Control of Splicing: Implications for the Study of Complex Traits
Source: PLoS Biol. 2008 Dec 23;6(12):e1000001. doi: 10.1371/journal.pbio.1000001 (PMC2605930; doi:10.1371/journal.pbio.1000001)
Supplement: Table S3 — (4.3 MB RTF) [file pbio.1000001.st003.rtf]

Table S3.   Lower confidence cis-acting SNPs that were shown in this study to influence exon level expression.  					
													
TISSUE TYPE	Probeset ID‡	LEVELa	Exon IDc	Transcript IDc	EXON gene/transcript ID	Chr	SNP assoc (sQTL)	MAF	SNP in probeset	Cross hybridization scored	p value	p value in other tissue typeb	
BRAIN	2342222	full	16747	2342220	TNNI3K	1	rs518769	0.174	N	1	3.17E-12	NS	
BRAIN	2342221	full	16747	2342220	TNNI3K	1	rs518769	0.174	N	1	2.40E-10	1.29E-04	
BRAIN	2351017	core	22205	2351004	GSTM5	1	rs10735234	0.382	N	1	3.19E-10	NS	
BRAIN	2351027	core	22206	2351004	GSTM5	1	rs10735234	0.382	N	1	4.51E-10	0.002	
BRAIN	2359264	extended	27118	2359243	LOC339400	1	rs12130219	0.277	N	1	2.19E-11	NS	
BRAIN	2376679	extended	37853	2376678	-	1	rs708727	0.43	Y	1	2.46E-23	1.58E-08	
BRAIN	2386896	extended	44114	2386867	LGALS8	1	rs766124	0.364	Y	1	4.51E-11	5.95E-06	
BRAIN	2409709	full	57948	2409708	GENSCAN00000036602	1	rs1417371	0.059	Y	1	2.92E-10	NS	
BRAIN	2412596	core	59724	2412529	NRD1	1	rs10888734	0.462	N	1	1.81E-11	1.42E-08	
BRAIN	2433517	full	72675	2433501	AK091688	1	rs515565	0.07	Y	2	1.46E-11	NS	
BRAIN	2452601	extended	84013	2452571	ELK4	1	rs2185811	0.097	N	1	1.68E-11	1.86E-04	
BRAIN	2456787	core	86653	2456746	EPRS	1	rs2289191	0.075	Y	1	1.43E-14	1.07E-05	
BRAIN	2467892	full	93603	2467855	SOX11	2	rs1429219	0.054	N	1	2.45E-10	NS	
BRAIN	2473078	core	96958	2473026	FLJ30851	2	rs2303291	0.247	N	1	1.79E-10	NS	
BRAIN	2473076	core	96956	2473026	FLJ30851	2	rs2288072	0.398	N	1	3.46E-10	NS	
BRAIN	2475386	extended	98382	2475348	LOC165186	2	rs10197378	0.242	N	1	5.46E-10	0.007	
BRAIN	2477122	full	99558	2477073	CRIM1	2	rs848609	0.048	N	1	4.45E-13	NS	
BRAIN	2480697	extended	101835	2480695	-	2	rs11690921	0.317	N	1	5.76E-12	7.85E-06	
BRAIN	2480982	core	102020	2480961	TACSTD1	2	rs4953495	0.054	N	2	8.37E-13	NS	
BRAIN	2480974	core	102015	2480961	TACSTD1	2	rs4953495	0.054	N	1	2.19E-10	NS	
BRAIN	2481470	full	102351	2481469	-	2	rs10205982	0.043	N	1	1.27E-10	NS	
BRAIN	2488086	core	106550	2488078	MPHOSPH10	2	rs357741	0.253	N	2	3.37E-12	1.68E-09	
BRAIN	2506225	extended	117762	2506204	SMPD4	2	rs6709245	0.36	N	2	5.14E-15	1.58E-12	
BRAIN	2515323	extended	123670	2515276	DYNC1I2	2	rs6754817	0.306	N	1	2.84E-11	1.27E-05	
BRAIN	2527306	extended	131232	2527305	-	2	rs2541403	0.478	N	1	1.68E-10	NS	
BRAIN	2528912	extended	132135	2528898	BC043385	2	rs10210670	0.382	N	1	8.41E-15	NS	
BRAIN	2533764	core	135162	2533670	CENTG2	2	rs2675136	0.301	N	1	4.80E-11	NS	
BRAIN	2541321	full	139917	2541230	NAG	2	rs11676115	0.339	N	2	3.15E-13	3.16E-07	
BRAIN	2565924	core	155403	2565902	KIAA1641	2	rs11691779	0.391	N	2	1.75E-10	1.36E-05	
BRAIN	2565949	extended	155421	2565935	KIAA1641	2	rs11691779	0.331	N	2	4.70E-34	6.49E-26	
BRAIN	2565945	extended	155417	2565935	KIAA1641	2	rs11691779	0.331	N	2	2.42E-31	9.52E-23	
BRAIN	2565976	core	155446	2565935	KIAA1641	2	rs11691779	0.391	N	2	5.00E-14	6.95E-08	
BRAIN	2565956	extended	155427	2565935	KIAA1641	2	rs11691779	0.391	N	2	5.91E-10	9.12E-06	
BRAIN	2571102	core	158639	2571075	ANAPC1	2	rs3814026	0.446	N	2	1.12E-14	5.11E-11	
BRAIN	2576557	extended	161917	2576554	FAM128A	2	rs6731616	0.097	N	2	3.37E-13	2.06E-07	
BRAIN	2576558	extended	161917	2576554	FAM128A	2	rs6731616	0.097	N	3	1.14E-11	6.13E-08	
BRAIN	2576556	extended	161916	2576554	FAM128A	2	rs6731616	0.097	N	3	3.21E-11	1.38E-06	
BRAIN	2594762	extended	173677	2594758	-	2	rs10931936	0.269	N	1	1.92E-11	NS	
BRAIN	2594765	extended	173679	2594758	-	2	rs10931936	0.269	N	1	1.01E-10	NS	
BRAIN	2618432	free	188705	2618407	MOBP	3	rs6797988	0.054	N	1	1.65E-13	NS	
BRAIN	2627326	full	194098	2627325	-	3	rs2887081	0.375	Y	1	3.17E-10	NS	
BRAIN	2647783	extended	206973	2647742	EIF2A	3	rs7631671	0.147	Y	1	4.39E-12	3.47E-11	
BRAIN	2650104	full	208470	2650100	LOC730109	3	rs665744	0.043	Y	1	9.92E-12	NS	
BRAIN	2655129	core	211673	2655113	KLHL24	3	rs12630877	0.242	N	1	2.69E-10	NS	
BRAIN	2657307	extended	212956	2657250	LPP	3	rs6787621	0.312	N	1	2.95E-18	4.23E-13	
BRAIN	2663054	extended	216585	2663038	C3orf31	3	rs6442290	0.457	Y	1	1.25E-11	0.008	
BRAIN	2663093	ambiguous	216608	2663092	C3orf31	3	rs1846206	0.426	N	1	2.09E-10	3.70E-04	
BRAIN	2665491	core	218175	2665472	EFHB	3	rs4103004	0.366	N	1	2.05E-10	NS	
BRAIN	2712400	full	247582	2712236	MUC4	3	rs2258447	0.048	N	1	4.74E-11	NS	
BRAIN	2729724	extended	258323	2729703	AJ617629	4	rs1843593	0.151	N	1	4.37E-10	9.86E-06	
BRAIN	2730837	full	259013	2730830	-	4	rs1851024	0.043	N	1	1.07E-12	NS	
BRAIN	2735361	extended	261834	2735341	BC027846	4	rs17013978	0.054	Y	1	3.99E-10	NS	
BRAIN	2750405	full	271366	2750395	GENSCAN00000002038	4	rs10008702	0.042	N	1	2.24E-10	NS	
BRAIN	2777053	core	287991	2777044	HSD17B13	4	rs10433937	0.274	N	1	1.60E-11	NS	
BRAIN	2777159	core	288064	2777113	SPARCL1	4	rs2011326	0.22	Y	2	8.95E-13	NS	
BRAIN	2780954	full	290468	2780907	DKK2	4	rs17037473	0.043	Y	1	6.63E-13	NS	
BRAIN	2798173	extended	301480	2798172	-	4	rs4863140	0.048	N	1	1.49E-12	0.001	
BRAIN	2798177	full	301482	2798176	GENSCAN00000013980	4	rs4863140	0.048	N	1	3.11E-19	1.19E-12	
BRAIN	2798179	extended	301483	2798178	-	4	rs4863140	0.048	N	1	5.97E-21	9.23E-16	
BRAIN	2807978	extended	307612	2807949	GHR	5	rs13182117	0.177	N	1	2.61E-10	NS	
BRAIN	2819256	extended	314824	2819235	BC047059	5	rs10452479	0.253	N	1	2.25E-20	4.56E-06	
BRAIN	2819255	extended	314824	2819235	BC047059	5	rs10452479	0.253	Y	2	6.72E-17	2.35E-05	
BRAIN	2819254	extended	314823	2819235	BC047059	5	rs10452479	0.253	N	2	3.22E-12	NS	
BRAIN	2843906	extended	330027	2843905	-	5	rs4700752	0.403	N	1	3.17E-12	NS	
BRAIN	2884272	extended	355240	2884270	-	5	rs6556396	0.317	N	1	1.38E-10	0.006	
BRAIN	2888546	core	357935	2888519	UIMC1	5	rs365132	0.484	Y	1	3.16E-13	1.21E-06	
BRAIN	2892473	extended	360189	2892453	C6orf86	6	rs4959793	0.179	Y	1	1.08E-12	6.45E-06	
BRAIN	2902572	core	366193	2902559	CSNK2B	6	rs805274	0.269	Y	1	1.68E-15	1.58E-07	
BRAIN	2903447	full	366651	2903435	HLA-DPB2	6	rs756440	0.231	N	1	3.55E-10	NS	
BRAIN	2912873	extended	372447	2912860	C6orf57	6	rs6455371	0.366	Y	1	3.38E-11	NS	
BRAIN	2914835	extended	373720	2914820	BCKDHB	6	rs9343974	0.484	Y	1	2.99E-10	0.01	
BRAIN	2923998	full	379411	2923984	RLBP1L2	6	rs9385283	0.269	Y	1	2.61E-12	NS	
BRAIN	2930583	extended	383645	2930568	BC041998	6	rs17725371	0.328	N	1	2.61E-11	NS	
BRAIN	2930931	extended	383860	2930907	LRP11	6	rs7752089	0.375	N	1	2.13E-10	NS	
BRAIN	2948901	core	395042	2948887	HLA-C	6	rs6457374	0.269	Y	1	1.07E-19	5.98E-15	
BRAIN	2959063	extended	401152	2959039	KHDRBS2	6	rs7758620	0.419	N	1	1.04E-10	0.049	
BRAIN	2993356	full	422570	2993352	GENSCAN00000035740	7	rs6966737	0.059	N	1	1.43E-11	NS	
BRAIN	3003060	extended	428698	3003042	LOC442676	7	rs1113765	0.215	N	3	2.93E-12	2.86E-10	
BRAIN	3004746	full	429643	3004745	AK130207	7	rs6978652	0.441	N	2	1.47E-10	NS	
BRAIN	3013855	full	435416	3013834	AK024600	7	rs9641200	0.043	N	1	2.73E-10	NS	
BRAIN	3036448	full	449399	3036447	GENSCAN00000003028	7	rs1001396	0.048	N	1	3.11E-10	NS	
BRAIN	3038126	extended	450394	3038065	ICA1	7	rs881451	0.048	Y	1	5.10E-11	NS	
BRAIN	3048179	extended	456700	3048134	C7orf44	7	rs11766602	0.167	N	1	1.07E-13	5.30E-09	
BRAIN	3053438	extended	459952	3053435	BC044608	7	rs4718180	0.403	Y	1	2.25E-18	8.33E-12	
BRAIN	3055297	full	461109	3055296	-	7	rs757839	0.097	N	1	1.66E-10	NS	
BRAIN	3067305	core	468531	3067302	LAMB1	7	rs10263341	0.366	Y	2	1.09E-10	0.018	
BRAIN	3069608	extended	470049	3069607	-	7	rs3808178	0.081	N	1	3.71E-10	NS	
BRAIN	3105938	core	493002	3105904	CPNE3	8	rs7000333	0.5	Y	1	4.04E-16	2.62E-12	
BRAIN	3107759	extended	494161	3107724	C8orf38	8	rs4735337	0.468	Y	2	8.67E-17	1.93E-05	
BRAIN	3122060	full	503185	3121751	CSMD1	8	rs12156233	0.433	Y	1	1.64E-11	NS	
BRAIN	3126019	extended	505659	3125993	FGL1	8	rs2653406	0.048	Y	1	1.91E-11	NS	
BRAIN	3140399	extended	514570	3140398	-	8	rs16938215	0.048	N	1	1.16E-11	NS	
BRAIN	3159863	full	526913	3159862	-	9	rs7868180	0.065	Y	1	6.19E-12	NS	
BRAIN	3180764	core	539671	3180717	C9orf102	9	rs12001	0.145	Y	1	7.12E-15	7.70E-07	
BRAIN	3185835	full	542818	3185832	-	9	rs10513208	0.043	N	1	1.35E-11	NS	
BRAIN	3188995	extended	544780	3188993	ARPC5L	9	rs628448	0.398	N	1	3.57E-13	0.01	
BRAIN	3199259	extended	551160	3199207	NFIB	9	rs2382453	0.339	Y	1	1.23E-10	NS	
BRAIN	3200715	core	552108	3200689	RPS6	9	rs957	0.096	Y	2	2.81E-11	2.09E-10	
BRAIN	3202451	extended	553138	3202450	GENSCAN00000011822	9	rs2282241	0.435	N	1	5.17E-10	NS	
BRAIN	3203812	core	553938	3203753	UBAP2	9	rs10971789	0.29	N	2	3.73E-11	4.92E-04	
BRAIN	3209179	extended	557145	3209060	TRPM3	9	rs12349882	0.043	N	1	4.21E-11	NS	
BRAIN	3210195	extended	557797	3210179	C9orf95	9	rs3752955	0.419	N	1	1.77E-10	1.36E-06	
BRAIN	3216046	extended	561453	3216023	C9orf130	9	rs12345187	0.301	N	1	1.13E-10	6.52E-10	
BRAIN	3235977	extended	573784	3235932	PRPF18	10	rs4748047	0.366	Y	1	1.95E-13	6.40E-04	
BRAIN	3235975	extended	573784	3235932	PRPF18	10	rs4748047	0.366	Y	1	3.54E-10	NS	
BRAIN	3243714	core	578630	3243708	BMS1	10	rs6593495	0.446	Y	2	1.80E-11	1.63E-09	
BRAIN	3250000	extended	582488	3249978	STOX1	10	rs2185653	0.194	Y	1	9.81E-11	NS	
BRAIN	3264818	full	591882	3264777	HABP2	10	rs11196383	0.043	Y	1	1.41E-11	NS	
BRAIN	3267737	core	593703	3267678	BRWD2	10	rs1652728	0.478	Y	1	9.09E-11	0.005	
BRAIN	3282083	extended	602768	3282072	C10orf51	10	rs7896781	0.364	N	3	6.55E-12	NS	
BRAIN	3291039	full	608393	3290875	ANK3	10	rs10761507	0.237	Y	1	1.81E-11	9.17E-05	
BRAIN	3303391	extended	616171	3303390	-	10	rs7072367	0.043	Y	1	6.36E-11	4.40E-07	
BRAIN	3303393	core	616172	3303392	BLOC1S2	10	rs12782963	0.043	N	1	2.82E-10	2.79E-04	
BRAIN	3307005	full	618404	3306984	GPAM	10	rs10749108	0.478	N	1	2.48E-12	NS	
BRAIN	3309367	core	619955	3309345	SFXN4	10	rs2275111	0.5	N	1	4.14E-11	8.91E-06	
BRAIN	3322820	core	628173	3322807	LDHC	11	rs2643856	0.11	Y	1	2.98E-16	3.92E-12	
BRAIN	3322819	core	628172	3322807	LDHC	11	rs2643856	0.11	N	1	4.51E-11	1.02E-06	
BRAIN	3349358	extended	644182	3349293	NCAM1	11	rs2850303	0.409	Y	2	1.59E-10	0.01	
BRAIN	3354667	full	647452	3354666	GENSCAN00000012571	11	rs11219981	0.226	N	1	2.22E-25	NS	
BRAIN	3364726	extended	653587	3364722	RPS13	11	rs1541533	0.062	N	1	1.41E-21	1.65E-21	
BRAIN	3366298	full	654496	3366297	GENSCAN00000005600	11	rs7128766	0.075	N	1	2.25E-10	NS	
BRAIN	3369263	extended	656363	3369249	APIP	11	rs10488802	0.323	Y	1	4.46E-10	2.47E-05	
BRAIN	3369269	extended	656368	3369249	APIP	11	rs10488802	0.323	Y	1	4.95E-10	1.74E-04	
BRAIN	3372407	extended	658318	3372403	-	11	rs10838738	0.349	Y	1	6.27E-13	6.04E-09	
BRAIN	3384461	extended	665382	3384459	AL137429	11	rs552997	0.441	Y	1	4.33E-10	NS	
BRAIN	3391749	core	669898	3391724	TMPRSS5	11	rs3902836	0.22	Y	1	9.10E-12	NS	
BRAIN	3391756	core	669901	3391724	TMPRSS5	11	rs1318296	0.156	Y	1	2.76E-11	NS	
BRAIN	3391757	core	669902	3391724	TMPRSS5	11	rs1318296	0.156	N	1	5.15E-10	NS	
BRAIN	3398491	extended	673926	3398482	SNX19	11	rs12418278	0.183	Y	1	6.25E-14	4.12E-13	
BRAIN	3398495	extended	673928	3398482	SNX19	11	rs12418278	0.183	Y	1	2.76E-10	4.57E-10	
BRAIN	3402642	core	676616	3402625	GAPDH	12	rs1060619	0.223	Y	2	4.14E-15	1.59E-08	
BRAIN	3404376	full	677637	3404371	OVOS2	12	rs7966062	0.462	N	3	7.65E-13	2.70E-04	
BRAIN	3409337	core	680784	3409330	MRPS35	12	rs2052673	0.194	Y	1	4.24E-12	8.71E-04	
BRAIN	3423519	ambiguous	689341	3423518	GENSCAN00000044904	12	rs10859297	0.458	Y	1	4.16E-11	NS	
BRAIN	3429357	extended	693034	3429312	HSP90B1	12	rs2722191	0.308	Y	1	2.55E-15	9.01E-04	
BRAIN	3443657	extended	701891	3443640	LOC731158	12	rs7966062	0.462	N	3	1.08E-13	NS	
BRAIN	3443651	extended	701888	3443640	LOC731158	12	rs7966062	0.462	N	2	1.53E-13	NS	
BRAIN	3443654	extended	701888	3443640	LOC731158	12	rs7966062	0.462	Y	3	4.15E-13	NS	
BRAIN	3443649	extended	701887	3443640	LOC731158	12	rs7966062	0.462	Y	2	2.24E-12	NS	
BRAIN	3443656	extended	701890	3443640	LOC731158	12	rs7966062	0.462	N	3	4.14E-11	NS	
BRAIN	3443928	extended	702039	3443924	uc001qwo.1	12	rs10505741	0.065	N	1	2.51E-19	7.41E-05	
BRAIN	3443927	extended	702038	3443924	uc001qwo.1	12	rs10505741	0.065	Y	1	9.66E-18	4.81E-05	
BRAIN	3444542	core	702380	3444540	TAS2R43	12	rs10772420	0.478	N	3	1.12E-14	6.36E-09	
BRAIN	3451845	full	706936	3451814	NELL2	12	rs1901556	0.392	N	1	5.41E-17	0.025	
BRAIN	3456337	full	709551	3456313	ATP5G2	12	rs1971762	0.317	Y	1	7.67E-17	0.002	
BRAIN	3456335	full	709549	3456313	ATP5G2	12	rs1971762	0.317	Y	1	1.98E-11	5.98E-04	
BRAIN	3464935	core	714763	3464912	WDR51B	12	rs10777177	0.468	N	2	4.75E-13	1.25E-12	
BRAIN	3488728	extended	729723	3488727	-	13	rs6314	0.086	Y	1	3.23E-10	NS	
BRAIN	3499556	extended	736566	3499453	TPP2	13	rs680840	0.441	Y	1	1.23E-12	1.56E-10	
BRAIN	3503501	full	739042	3503376	MRPL3	13	rs1806658	0.371	N	1	3.73E-10	NS	
BRAIN	3509655	core	742888	3509645	SOHLH2	13	rs943895	0.473	N	2	5.44E-11	NS	
BRAIN	3510021	extended	743118	3510018	GENSCAN00000066481	13	rs1324030	0.059	Y	1	5.53E-10	NS	
BRAIN	3516273	full	747084	3516228	PCDH20	13	rs2876807	0.043	N	2	1.46E-10	NS	
BRAIN	3521542	extended	750347	3521484	UGCGL2	13	rs12876018	0.323	N	1	2.62E-12	NS	
BRAIN	3528305	extended	754408	3528172	TRA@	14	rs1534813	0.048	N	1	1.65E-12	NS	
BRAIN	3531629	extended	756361	3531553	AKAP6	14	rs7157533	0.43	Y	1	1.92E-11	NS	
BRAIN	3537457	full	759931	3537455	LOC645798	14	rs1189816	0.043	N	1	1.81E-10	NS	
BRAIN	3553713	extended	770166	3553690	MARK3	14	rs12879663	0.355	Y	1	6.70E-12	2.08E-10	
BRAIN	3562321	full	775195	3562320	GENSCAN00000003902	14	rs17181608	0.188	N	1	1.67E-21	NS	
BRAIN	3581330	ambiguous	787010	3581329	AHNAK2	14	rs2841277	0.43	Y	3	6.17E-12	NS	
BRAIN	3581366	ambiguous	787028	3581365	AHNAK2	14	rs1048257	0.419	Y	1	1.33E-10	NS	
BRAIN	3583729	extended	788432	3583728	-	15	rs7402104	0.065	N	1	2.83E-10	NS	
BRAIN	3584221	full	788723	3584200	GENSCAN00000055978	15	rs12904856	0.156	N	3	2.04E-12	NS	
BRAIN	3584234	full	788734	3584200	GENSCAN00000055978	15	rs1878504	0.28	N	1	1.60E-10	NS	
BRAIN	3584254	extended	788748	3584251	-	15	rs12904856	0.156	Y	1	4.67E-11	NS	
BRAIN	3584270	full	788760	3584262	GENSCAN00000022013	15	rs12904329	0.242	N	3	9.48E-12	NS	
BRAIN	3584360	extended	788825	3584358	BC034815	15	rs12904329	0.242	N	3	7.69E-11	NS	
BRAIN	3584366	extended	788830	3584358	BC034815	15	rs12904329	0.242	N	1	1.84E-10	NS	
BRAIN	3588560	full	791376	3588559	-	15	rs12438903	0.043	Y	1	2.91E-10	NS	
BRAIN	3595408	extended	795619	3595402	-	15	rs1567620	0.435	N	2	3.52E-13	NS	
BRAIN	3599526	full	798171	3599495	CORO2B	15	rs2924634	0.048	N	1	1.60E-10	NS	
BRAIN	3641853	full	824500	3641823	PRKXP1	15	rs4965320	0.376	Y	3	1.58E-10	6.12E-09	
BRAIN	3641836	full	824492	3641823	PRKXP1	15	rs4965320	0.376	Y	3	4.41E-10	1.08E-08	
BRAIN	3650480	free	829689	3650481	-	16	rs6498721	0.043	N	1	5.62E-13	NS	
BRAIN	3659476	extended	834882	3659475	-	16	rs12597569	0.371	N	1	2.38E-11	NS	
BRAIN	3670133	extended	841400	3670132	-	16	rs4888911	0.253	N	1	2.61E-10	0.003	
BRAIN	3674918	core	844375	3674886	C16orf35	16	rs183350	0.12	N	1	2.40E-10	9.18E-04	
BRAIN	3676813	core	845463	3676763	ABCA3	16	rs11867129	0.124	Y	1	1.11E-10	1.92E-04	
BRAIN	3692924	extended	855056	3692923	-	16	rs9933064	0.054	N	1	1.46E-13	NS	
BRAIN	3712429	core	866876	3712363	M-RIP	17	rs4985741	0.43	N	3	7.96E-14	0.036	
BRAIN	3723865	extended	873599	3723864	-	17	rs2532269	0.211	Y	1	6.65E-12	1.07E-09	
BRAIN	3723898	core	873615	3723891	LRRC37A	17	rs2532329	0.191	Y	2	1.57E-13	0.025	
BRAIN	3723924	core	873627	3723891	LRRC37A	17	rs2668692	0.213	Y	2	4.07E-13	2.62E-07	
BRAIN	3723933	core	873630	3723891	LRRC37A	17	rs2668692	0.213	N	3	2.62E-12	0.004	
BRAIN	3723912	core	873622	3723891	LRRC37A	17	rs2532329	0.191	N	2	2.80E-12	5.58E-11	
BRAIN	3723900	core	873615	3723891	LRRC37A	17	rs2668692	0.213	Y	2	4.48E-12	NS	
BRAIN	3723922	core	873626	3723891	LRRC37A	17	rs2668692	0.213	Y	3	1.61E-11	0.008	
BRAIN	3723914	core	873623	3723891	LRRC37A	17	rs2532329	0.191	N	2	2.26E-11	2.13E-07	
BRAIN	3723897	core	873615	3723891	LRRC37A	17	rs2532329	0.191	Y	2	2.31E-11	0.012	
BRAIN	3723896	core	873615	3723891	LRRC37A	17	rs2532329	0.191	N	3	2.47E-11	0.032	
BRAIN	3723915	extended	873623	3723891	LRRC37A	17	rs2668692	0.213	N	1	9.22E-11	0.005	
BRAIN	3723902	core	873615	3723891	LRRC37A	17	rs2668692	0.213	N	3	1.08E-10	NS	
BRAIN	3723894	core	873614	3723891	LRRC37A	17	rs2532329	0.191	N	3	1.20E-10	0.002	
BRAIN	3723895	core	873614	3723891	LRRC37A	17	rs2668692	0.213	N	2	1.77E-10	0.012	
BRAIN	3724616	extended	874036	3724591	C17orf57	17	rs7214410	0.419	Y	1	4.20E-10	0.012	
BRAIN	3740057	full	883484	3739962	ABR	17	rs2083811	0.14	Y	3	3.73E-11	5.07E-04	
BRAIN	3748411	core	888477	3748400	LOC220594	17	rs3907609	0.489	Y	2	9.33E-12	0.002	
BRAIN	3759961	ambiguous	895235	3759960	-	17	rs393152	0.231	N	3	1.79E-11	0.039	
BRAIN	3759965	ambiguous	895237	3759964	LRRC37A2	17	rs393152	0.231	Y	2	3.64E-14	0.001	
BRAIN	3759969	ambiguous	895239	3759968	LRRC37A2	17	rs393152	0.231	N	2	5.26E-12	0.024	
BRAIN	3759973	full	895241	3759970	LRRC37A2	17	rs393152	0.231	N	3	3.78E-16	3.35E-05	
BRAIN	3759972	full	895241	3759970	LRRC37A2	17	rs393152	0.231	N	2	1.95E-14	0.018	
BRAIN	3759971	full	895240	3759970	LRRC37A2	17	rs393152	0.231	N	2	8.50E-14	9.48E-05	
BRAIN	3759975	ambiguous	895242	3759974	LRRC37A2	17	rs393152	0.231	Y	3	4.84E-13	0.002	
BRAIN	3759978	extended	895243	3759976	LRRC37A3	17	rs417968	0.285	N	3	1.07E-10	NS	
BRAIN	3759985	extended	895245	3759982	-	17	rs393152	0.231	N	2	1.34E-11	NS	
BRAIN	3759987	extended	895246	3759986	-	17	rs393152	0.231	Y	3	4.68E-10	0.02	
BRAIN	3760011	full	895258	3760010	-	17	rs393152	0.269	N	2	1.56E-23	2.72E-23	
BRAIN	3760018	extended	895262	3760013	BC019018	17	rs393152	0.231	N	3	8.18E-17	4.28E-16	
BRAIN	3760031	extended	895267	3760013	BC019018	17	rs393152	0.231	N	2	1.26E-11	3.08E-09	
BRAIN	3760026	extended	895264	3760013	BC019018	17	rs393152	0.231	N	3	1.79E-11	3.68E-05	
BRAIN	3760188	extended	895377	3760137	KIAA1267	17	rs2532269	0.211	N	1	3.03E-15	1.44E-05	
BRAIN	3760190	extended	895379	3760137	KIAA1267	17	rs1981997	0.215	Y	1	9.27E-14	2.22E-05	
BRAIN	3760192	extended	895381	3760137	KIAA1267	17	rs1981997	0.215	Y	1	1.24E-13	1.12E-06	
BRAIN	3760205	extended	895392	3760137	KIAA1267	17	rs1981997	0.215	N	1	1.30E-12	0.009	
BRAIN	3760191	extended	895380	3760137	KIAA1267	17	rs1981997	0.215	Y	1	3.30E-12	1.85E-06	
BRAIN	3760204	extended	895391	3760137	KIAA1267	17	rs1981997	0.215	Y	1	5.03E-12	0.013	
BRAIN	3760197	full	895385	3760137	KIAA1267	17	rs2532269	0.211	N	2	1.39E-11	6.44E-07	
BRAIN	3760213	core	895399	3760137	KIAA1267	17	rs1981997	0.215	N	1	1.44E-11	2.92E-06	
BRAIN	3760210	extended	895397	3760137	KIAA1267	17	rs1981997	0.215	N	1	3.46E-11	0.018	
BRAIN	3760193	extended	895382	3760137	KIAA1267	17	rs1981997	0.215	N	1	1.49E-10	3.77E-04	
BRAIN	3760211	extended	895398	3760137	KIAA1267	17	rs1981997	0.215	Y	1	5.91E-10	0.008	
BRAIN	3760249	extended	895423	3760247	-	17	rs2668692	0.213	Y	1	1.44E-10	2.52E-06	
BRAIN	3760283	extended	895440	3760268	ARL17P1	17	rs2532329	0.191	N	1	6.66E-17	4.47E-11	
BRAIN	3760288	full	895441	3760268	ARL17P1	17	rs2532329	0.191	N	1	1.05E-16	1.28E-05	
BRAIN	3760285	core	895440	3760268	ARL17P1	17	rs2532329	0.191	N	2	3.85E-16	7.96E-11	
BRAIN	3760279	full	895439	3760268	ARL17P1	17	rs2532329	0.191	N	3	7.06E-16	2.74E-10	
BRAIN	3760278	extended	895438	3760268	ARL17P1	17	rs2532329	0.191	Y	2	1.04E-15	3.85E-12	
BRAIN	3760286	core	895440	3760268	ARL17P1	17	rs2532329	0.191	Y	2	4.17E-14	2.11E-08	
BRAIN	3760281	full	895439	3760268	ARL17P1	17	rs2532329	0.191	Y	2	4.76E-12	7.87E-12	
BRAIN	3760276	extended	895437	3760268	ARL17P1	17	rs2668692	0.213	Y	2	4.05E-11	2.15E-06	
BRAIN	3760275	extended	895437	3760268	ARL17P1	17	rs2532329	0.191	Y	1	1.30E-10	1.35E-07	
BRAIN	3760287	full	895440	3760268	ARL17P1	17	rs2532329	0.191	N	3	3.33E-10	2.06E-06	
BRAIN	3779862	core	907351	3779817	CEP192	18	rs1786263	0.349	Y	1	3.17E-12	0.037	
BRAIN	3788894	full	913115	3788874	C18orf54	18	rs1523868	0.237	Y	1	4.44E-10	2.76E-07	
BRAIN	3793987	extended	916281	3793986	-	18	rs522009	0.253	Y	1	3.43E-12	NS	
BRAIN	3795490	extended	917284	3795466	C18orf22	18	rs12606223	0.326	N	1	1.61E-11	NS	
BRAIN	3801754	extended	921217	3801753	-	18	rs1945168	0.355	N	1	1.14E-10	NS	
BRAIN	3802867	full	921965	3802866	-	18	rs16947857	0.043	Y	1	1.31E-13	NS	
BRAIN	3805155	full	923443	3805117	AK128175	18	rs10084057	0.076	Y	1	4.49E-10	NS	
BRAIN	3808879	full	925836	3808854	TCF4	18	rs13381800	0.258	Y	1	2.29E-10	NS	
BRAIN	3813483	extended	928688	3813482	ENST00000382649	18	rs6566816	0.226	Y	1	7.96E-11	0.025	
BRAIN	3831808	extended	939406	3831806	BC041478	19	rs320881	0.271	N	1	1.66E-15	NS	
BRAIN	3840207	extended	944238	3840194	LOC400713	19	rs2059818	0.435	Y	1	1.49E-12	NS	
BRAIN	3857115	core	953773	3857105	ZNF91	19	rs1020075	0.225	Y	1	7.89E-12	2.76E-10	
BRAIN	3864711	extended	958174	3864709	-	19	rs417400	0.489	Y	1	1.60E-11	0.002	
BRAIN	3872028	ambiguous	962255	3872027	BC37295_3	19	rs2052242	0.435	Y	1	2.82E-10	0.016	
BRAIN	3872280	core	962393	3872274	VN1R1	19	rs11084499	0.272	N	2	5.00E-10	5.09E-06	
BRAIN	3873654	core	963130	3873629	SIRPA	20	rs6075340	0.369	Y	1	2.21E-27	3.99E-20	
BRAIN	3874507	core	963675	3874498	VISA	20	rs8116776	0.242	Y	2	2.48E-12	7.11E-04	
BRAIN	3879393	core	966753	3879372	C20orf19	20	rs6035837	0.281	Y	1	1.50E-12	2.40E-11	
BRAIN	3883401	extended	969209	3883382	ERGIC3	20	rs2104417	0.167	N	1	1.61E-10	0.006	
BRAIN	3890845	core	973762	3890840	C20orf85	20	rs6513367	0.048	N	1	5.22E-10	NS	
BRAIN	3894800	extended	976139	3894790	LOC441938	20	rs6075340	0.369	Y	1	6.37E-24	4.46E-12	
BRAIN	3894839	extended	976168	3894836	-	20	rs58532	0.489	N	1	5.35E-10	NS	
BRAIN	3905366	extended	982464	3905332	SNORA71A	20	rs752774	0.194	Y	1	5.99E-16	6.69E-05	
BRAIN	3905360	extended	982464	3905332	SNORA71A	20	rs752774	0.194	N	1	4.36E-15	4.75E-06	
BRAIN	3905362	extended	982464	3905332	SNORA71A	20	rs752774	0.194	N	1	4.07E-12	1.44E-05	
BRAIN	3905358	extended	982464	3905332	SNORA71A	20	rs752774	0.194	N	1	1.35E-11	8.91E-06	
BRAIN	3906445	full	983150	3906390	PTPRT	20	rs6030745	0.043	N	1	4.88E-11	NS	
BRAIN	3947321	extended	1007914	3947310	C22orf32	22	rs2284087	0.441	Y	2	2.40E-11	7.37E-07	
BRAIN	3948555	core	1008693	3948543	FAM118A	22	rs104664	0.102	Y	1	1.66E-12	3.54E-08	
PBMC	2325839	extended	6635	2325836	-	1	rs3091242	0.5	Y	1	4.38E-11	0.002	
PBMC	2325837	extended	6635	2325836	-	1	rs3091242	0.5	N	3	6.04E-11	0.023	
PBMC	2325985	extended	6713	2325984	AK091825	1	rs3091242	0.5	Y	3	4.74E-10	0.018	
PBMC	2331628	extended	10083	2331602	PPIE	1	rs7547787	0.312	Y	1	1.08E-19	1.27E-06	
PBMC	2331732	extended	10140	2331727	CAP1	1	rs3806206	0.125	Y	1	5.35E-18	NS	
PBMC	2336593	core	13080	2336585	SCP2	1	rs11206043	0.387	N	1	5.77E-11	NS	
PBMC	2336600	core	13086	2336585	SCP2	1	rs11206043	0.387	N	1	2.58E-10	NS	
PBMC	2336595	core	13081	2336585	SCP2	1	rs11206043	0.387	N	1	2.87E-10	NS	
PBMC	2336596	core	13082	2336585	SCP2	1	rs11206043	0.387	N	1	2.89E-10	NS	
PBMC	2337030	extended	13340	2337003	MRPL37	1	rs10888838	0.144	Y	1	5.16E-11	0.003	
PBMC	2338493	full	14259	2338487	FLJ10986	1	rs2143593	0.05	N	1	2.22E-10	NS	
PBMC	2345538	full	18831	2345507	GENSCAN00000033752	1	rs10489945	0.044	N	1	2.48E-10	NS	
PBMC	2345669	core	18924	2345617	PKN2	1	rs786918	0.4	N	1	5.20E-11	1.65E-04	
PBMC	2363005	core	29149	2362991	CASQ1	1	rs10797062	0.244	N	1	3.70E-11	NS	
PBMC	2363533	core	29424	2363525	NDUFS2	1	rs3813620	0.05	Y	1	8.72E-11	1.67E-04	
PBMC	2385708	core	43391	2385696	C1orf57	1	rs1555393	0.244	N	1	5.63E-10	0.029	
PBMC	2386903	extended	44118	2386867	LGALS8	1	rs2243527	0.219	Y	1	3.91E-11	2.72E-05	
PBMC	2389398	full	45788	2389247	KIF26B	1	rs12120810	0.044	N	1	5.97E-10	NS	
PBMC	2393824	extended	48185	2393816	C1orf174	1	rs7367066	0.312	Y	1	6.82E-12	5.21E-04	
PBMC	2395133	core	49062	2395123	UTS2	1	rs161811	0.044	Y	1	3.72E-17	NS	
PBMC	2395128	core	49059	2395123	UTS2	1	rs161811	0.044	N	2	8.88E-12	NS	
PBMC	2398829	core	51296	2398820	PADI2	1	rs2014725	0.456	N	1	1.92E-11	NS	
PBMC	2398823	core	51292	2398820	PADI2	1	rs2014725	0.456	N	1	3.32E-11	NS	
PBMC	2398837	core	51304	2398820	PADI2	1	rs2014725	0.456	N	1	6.50E-11	NS	
PBMC	2398844	core	51310	2398820	PADI2	1	rs2014725	0.456	N	1	1.93E-10	NS	
PBMC	2398825	core	51294	2398820	PADI2	1	rs2014725	0.456	N	1	2.56E-10	NS	
PBMC	2398845	core	51311	2398820	PADI2	1	rs2014725	0.456	N	1	3.68E-10	NS	
PBMC	2402146	extended	53339	2402111	C1orf63	1	rs3091242	0.5	Y	3	4.26E-11	6.83E-04	
PBMC	2402148	extended	53339	2402111	C1orf63	1	rs3091242	0.5	Y	3	8.26E-11	3.92E-06	
PBMC	2402155	extended	53344	2402153	-	1	rs3091242	0.5	Y	2	9.49E-12	0.003	
PBMC	2404524	core	54799	2404521	PEF1	1	rs4949455	0.35	Y	3	2.55E-10	4.04E-06	
PBMC	2410224	extended	58258	2410218	LOC126661	1	rs1494813	0.375	N	1	2.43E-13	4.56E-07	
PBMC	2410227	extended	58261	2410218	LOC126661	1	rs1494813	0.375	N	2	4.28E-10	4.85E-06	
PBMC	2412601	core	59728	2412529	NRD1	1	rs10888740	0.419	N	1	9.40E-11	3.21E-06	
PBMC	2414960	core	61274	2414958	TACSTD2	1	rs6690139	0.169	Y	1	1.58E-12	NS	
PBMC	2414961	core	61274	2414958	TACSTD2	1	rs6690139	0.169	Y	1	2.45E-10	NS	
PBMC	2418472	core	63486	2418451	CRYZ	1	rs1409785	0.35	N	3	5.89E-12	5.12E-04	
PBMC	2418460	core	63480	2418451	CRYZ	1	rs1409785	0.35	Y	2	2.65E-11	1.23E-10	
PBMC	2418489	extended	63499	2418451	CRYZ	1	rs1409785	0.35	N	3	8.42E-11	8.01E-04	
PBMC	2421300	core	65258	2421271	15-Sep	1	rs4656125	0.119	Y	1	8.57E-12	4.60E-07	
PBMC	2421857	core	65629	2421843	GBP3	1	rs12120863	0.256	N	1	8.41E-11	0.003	
PBMC	2427213	core	68981	2427208	GSTM3	1	rs10735234	0.419	N	1	5.54E-10	1.00E-04	
PBMC	2428751	core	69862	2428699	PHTF1	1	rs1230661	0.262	Y	2	6.24E-12	6.94E-05	
PBMC	2434570	extended	73311	2434569	-	1	rs6587518	0.381	Y	1	1.62E-13	NS	
PBMC	2438924	core	75788	2438892	FCRL5	1	rs1412676	0.194	Y	1	2.28E-12	NS	
PBMC	2438895	core	75773	2438892	FCRL5	1	rs10489671	0.194	N	2	6.71E-12	NS	
PBMC	2438918	core	75785	2438892	FCRL5	1	rs1412676	0.194	Y	1	1.67E-11	NS	
PBMC	2438893	core	75772	2438892	FCRL5	1	rs1412676	0.194	Y	1	1.44E-10	NS	
PBMC	2438976	extended	75815	2438970	GENSCAN00000024614	1	rs10489671	0.194	N	3	1.20E-11	NS	
PBMC	2438978	full	75816	2438977	GENSCAN00000024614	1	rs1412676	0.194	N	3	2.27E-11	NS	
PBMC	2438983	full	75818	2438982	-	1	rs1412676	0.194	N	3	1.07E-12	NS	
PBMC	2438985	full	75819	2438984	GENSCAN00000024614	1	rs10489671	0.194	N	3	6.08E-10	0.014	
PBMC	2440485	core	76657	2440476	F11R	1	rs1062827	0.275	Y	1	4.96E-12	1.16E-06	
PBMC	2458559	core	87745	2458513	TMEM63A	1	rs1051741	0.094	Y	2	3.43E-10	NS	
PBMC	2464740	extended	91616	2464731	-	1	rs11799428	0.044	N	1	7.67E-12	NS	
PBMC	2465386	full	92049	2465324	AHCTF1	1	rs1613209	0.469	Y	2	1.05E-10	0.024	
PBMC	2466156	core	92449	2466141	ACP1	2	rs2290911	0.312	N	1	7.11E-11	7.75E-05	
PBMC	2473299	extended	97111	2473284	CENPO	2	rs4665716	0.05	N	2	5.82E-10	NS	
PBMC	2482320	extended	102887	2482316	ACYP2	2	rs1877908	0.381	N	1	8.83E-12	1.90E-05	
PBMC	2485366	extended	104855	2485334	GENSCAN00000007123	2	rs6750096	0.312	N	2	2.92E-13	9.10E-05	
PBMC	2488334	core	106716	2488252	DYSF	2	rs11691076	0.138	N	1	6.15E-11	NS	
PBMC	2488291	core	106683	2488252	DYSF	2	rs2542501	0.094	N	1	5.28E-10	NS	
PBMC	2489187	extended	107238	2489172	MTHFD2	2	rs895988	0.45	N	2	1.92E-11	0.008	
PBMC	2491522	extended	108722	2491514	-	2	rs1044973	0.488	N	1	3.81E-12	NS	
PBMC	2493006	extended	109580	2492968	FLJ40330	2	rs6715865	0.05	N	2	3.25E-13	NS	
PBMC	2493001	extended	109579	2492968	FLJ40330	2	rs6715865	0.05	N	1	2.45E-10	NS	
PBMC	2501324	extended	114679	2501317	LOC654433	2	rs4849179	0.344	N	1	8.65E-11	8.57E-04	
PBMC	2501326	extended	114680	2501317	LOC654433	2	rs4849179	0.344	N	1	4.32E-10	4.09E-04	
PBMC	2504348	core	116666	2504328	GYPC	2	rs6732145	0.369	N	2	1.00E-15	0.029	
PBMC	2508126	full	118944	2508123	LOC647012	2	rs6713376	0.044	N	1	6.60E-11	NS	
PBMC	2524224	core	129260	2524016	PARD3B	2	rs861066	0.131	N	1	2.14E-10	NS	
PBMC	2531414	extended	133645	2531377	SP100	2	rs1649890	0.312	N	1	1.73E-16	NS	
PBMC	2531386	extended	133630	2531377	SP100	2	rs1427283	0.056	N	2	1.48E-13	NS	
PBMC	2531387	extended	133631	2531377	SP100	2	rs1427283	0.056	N	1	7.85E-12	0.002	
PBMC	2534890	extended	135898	2534865	ASB1	2	rs3739070	0.062	N	3	1.57E-13	2.50E-10	
PBMC	2537140	core	137237	2537109	SH3YL1	2	rs2290911	0.312	N	2	5.95E-12	NS	
PBMC	2537141	core	137238	2537109	SH3YL1	2	rs2290911	0.312	N	1	2.81E-10	NS	
PBMC	2553703	core	147836	2553682	C2orf63	2	rs7349405	0.169	N	1	2.60E-11	2.80E-04	
PBMC	2553701	core	147835	2553682	C2orf63	2	rs7349405	0.169	N	1	2.72E-11	0.021	
PBMC	2553699	core	147833	2553682	C2orf63	2	rs7349405	0.169	N	1	3.53E-10	0.024	
PBMC	2556396	extended	149547	2556393	AL355732	2	rs12477412	0.294	N	1	2.62E-11	NS	
PBMC	2560475	extended	152003	2560474	AL832916	2	rs17563336	0.219	N	1	1.53E-13	0.003	
PBMC	2560479	extended	152005	2560478	-	2	rs12713820	0.446	N	1	4.28E-19	1.41E-11	
PBMC	2560480	extended	152006	2560478	-	2	rs17563336	0.219	N	1	4.24E-11	NS	
PBMC	2560482	full	152007	2560481	GENSCAN00000000999	2	rs7575416	0.387	N	1	2.46E-12	5.63E-07	
PBMC	2560491	extended	152011	2560490	-	2	rs12713820	0.38	N	1	4.05E-13	1.43E-07	
PBMC	2560493	extended	152012	2560492	-	2	rs13012537	0.4	N	1	1.67E-13	9.21E-06	
PBMC	2566605	core	155829	2566586	TSGA10	2	rs2516835	0.425	N	1	1.10E-10	0.012	
PBMC	2571880	core	159099	2571874	MGC70863	2	rs4849261	0.394	N	2	2.84E-16	3.05E-04	
PBMC	2571894	core	159112	2571874	MGC70863	2	rs4849261	0.394	N	3	3.21E-13	4.62E-04	
PBMC	2588131	extended	169366	2588127	ATP5G3	2	rs11888507	0.181	N	1	7.84E-14	9.66E-14	
PBMC	2602988	extended	178990	2602901	TRIP12	2	rs6722477	0.138	N	1	9.43E-16	1.93E-06	
PBMC	2606607	core	181207	2606574	NDUFA10	2	rs958960	0.306	N	1	2.70E-10	2.02E-04	
PBMC	2607843	full	182005	2607757	CNTN6	3	rs17035505	0.044	N	1	8.74E-11	NS	
PBMC	2612016	full	184641	2612012	C3orf20	3	rs1901	0.359	Y	1	2.86E-11	1.37E-08	
PBMC	2616183	core	187336	2616166	CRTAP	3	rs11707667	0.463	Y	1	4.11E-11	2.36E-06	
PBMC	2618308	free	188643	2618333	RPSA	3	rs4016652	0.325	Y	1	1.54E-10	NS	
PBMC	2622214	core	190963	2622196	APEH	3	rs3197999	0.4	Y	1	3.82E-20	6.73E-15	
PBMC	2626207	core	193348	2626167	PXK	3	rs11130638	0.375	Y	1	1.32E-10	2.43E-04	
PBMC	2632902	full	197633	2632832	EPHA6	3	rs9848435	0.044	Y	1	9.00E-11	NS	
PBMC	2636499	core	199871	2636483	SIDT1	3	rs7611694	0.412	Y	1	1.08E-11	NS	
PBMC	2663828	core	217069	2663810	XPC	3	rs4685078	0.244	Y	1	3.89E-10	4.36E-05	
PBMC	2670586	core	221356	2670481	ULK4	3	rs12635286	0.15	N	1	7.82E-11	0.003	
PBMC	2689664	extended	233388	2689516	ZBTB20	3	rs12639377	0.146	N	1	2.06E-17	8.18E-14	
PBMC	2705150	extended	243008	2705148	RPL22L1	3	rs3732927	0.325	N	1	2.41E-10	NS	
PBMC	2713843	core	248446	2713837	ZNF718	4	rs6830247	0.25	Y	1	1.69E-13	2.78E-08	
PBMC	2720198	extended	252338	2720181	MED28	4	rs1558377	0.425	N	1	1.79E-14	8.95E-12	
PBMC	2732874	core	260269	2732844	ANXA3	4	rs7661370	0.119	Y	1	3.59E-10	NS	
PBMC	2732881	core	260273	2732844	ANXA3	4	rs5945	0.081	N	1	5.89E-10	NS	
PBMC	2741276	extended	265705	2741236	USP53	4	rs13113462	0.116	N	1	8.69E-12	NS	
PBMC	2741263	extended	265696	2741236	USP53	4	rs13113462	0.116	N	1	1.89E-11	NS	
PBMC	2741266	extended	265698	2741236	USP53	4	rs13113462	0.116	Y	1	3.31E-10	0.026	
PBMC	2741278	extended	265706	2741236	USP53	4	rs13113462	0.116	Y	1	5.64E-10	NS	
PBMC	2745716	core	268471	2745712	LOC441046	4	rs1510881	0.206	N	1	3.10E-10	NS	
PBMC	2751938	core	272338	2751936	GALNT7	4	rs2877748	0.344	Y	1	1.44E-19	3.42E-08	
PBMC	2754587	extended	274044	2754582	SNX25	4	rs2310159	0.412	Y	1	3.97E-10	NS	
PBMC	2758314	core	276173	2758298	LRPAP1	4	rs6811423	0.213	Y	1	7.33E-12	4.64E-04	
PBMC	2758302	core	276168	2758298	LRPAP1	4	rs6811423	0.213	N	1	2.94E-10	0.002	
PBMC	2771679	core	284667	2771654	CENPC1	4	rs11250	0.371	Y	1	2.46E-25	4.84E-23	
PBMC	2774493	full	286388	2774492	-	4	rs10010725	0.05	Y	1	4.52E-10	NS	
PBMC	2777101	extended	288026	2777070	HSD17B11	4	rs6827817	0.5	Y	1	9.22E-12	1.19E-09	
PBMC	2794925	core	299436	2794902	AGA	4	rs4690523	0.45	N	1	4.16E-10	NS	
PBMC	2798585	extended	301727	2798584	-	5	rs7725227	0.15	Y	1	6.52E-11	0.006	
PBMC	2799111	extended	302056	2799108	-	5	rs401681	0.412	Y	1	9.82E-12	2.35E-04	
PBMC	2800489	core	302963	2800477	SRD5A1	5	rs7706809	0.431	Y	3	5.94E-15	1.25E-13	
PBMC	2808398	full	307871	2808397	-	5	rs17245133	0.056	N	1	5.58E-10	NS	
PBMC	2820610	full	315710	2820609	-	5	rs2017209	0.05	Y	1	1.30E-10	NS	
PBMC	2821249	core	316112	2821194	CAST	5	rs13362120	0.369	N	2	3.30E-26	1.05E-08	
PBMC	2827016	full	319733	2826996	GENSCAN00000064227	5	rs2029547	0.051	Y	1	1.14E-10	NS	
PBMC	2835808	core	324963	2835792	GM2A	5	rs1004736	0.162	N	1	2.00E-11	NS	
PBMC	2843076	core	329572	2843064	PRELID1	5	rs3733875	0.125	Y	2	3.11E-10	1.61E-06	
PBMC	2845740	core	331071	2845699	SLC12A7	5	rs4580814	0.488	N	3	6.16E-13	NS	
PBMC	2845744	core	331075	2845699	SLC12A7	5	rs4580814	0.488	Y	1	1.33E-12	NS	
PBMC	2845742	core	331073	2845699	SLC12A7	5	rs4580814	0.488	N	1	1.69E-11	NS	
PBMC	2845722	full	331056	2845699	SLC12A7	5	rs4580814	0.488	Y	3	5.38E-11	NS	
PBMC	2845741	core	331072	2845699	SLC12A7	5	rs4580814	0.488	Y	1	8.92E-11	NS	
PBMC	2845700	core	331041	2845699	SLC12A7	5	rs4580814	0.488	Y	1	1.16E-10	NS	
PBMC	2845719	core	331054	2845699	SLC12A7	5	rs4580814	0.488	Y	1	1.22E-10	NS	
PBMC	2845728	core	331062	2845699	SLC12A7	5	rs4580814	0.488	N	1	1.48E-10	NS	
PBMC	2845734	core	331066	2845699	SLC12A7	5	rs4580814	0.488	N	1	1.60E-10	NS	
PBMC	2855380	full	337305	2855368	FLJ32255	5	rs2548345	0.325	N	1	5.32E-10	NS	
PBMC	2856063	core	337718	2856044	EMB	5	rs6868380	0.331	N	3	2.04E-13	NS	
PBMC	2859681	core	339989	2859667	CENPK	5	rs154861	0.412	N	1	5.18E-11	NS	
PBMC	2859699	core	340002	2859667	CENPK	5	rs6897886	0.45	N	1	7.97E-11	NS	
PBMC	2859685	core	339992	2859667	CENPK	5	rs6897886	0.45	N	1	1.66E-10	0.006	
PBMC	2859702	core	340005	2859667	CENPK	5	rs380327	0.45	N	1	2.46E-10	NS	
PBMC	2859700	core	340003	2859667	CENPK	5	rs6897886	0.45	N	1	4.44E-10	NS	
PBMC	2864260	extended	342729	2864237	HOMER1	5	rs11948804	0.05	N	1	2.25E-10	NS	
PBMC	2865055	extended	343242	2865050	RPS23	5	rs226206	0.237	N	1	3.13E-10	7.50E-05	
PBMC	2868133	core	345194	2868131	ERAP1	5	rs13160562	0.356	Y	1	1.18E-17	0.004	
PBMC	2868139	extended	345199	2868131	ERAP1	5	rs13160562	0.356	N	1	3.50E-15	1.03E-06	
PBMC	2868203	extended	345247	2868131	ERAP1	5	rs1981846	0.475	Y	1	1.42E-12	NS	
PBMC	2870905	full	346911	2870889	C5orf13	5	rs3797730	0.056	N	1	5.95E-10	NS	
PBMC	2883622	extended	354809	2883609	CLINT1	5	rs10050757	0.275	Y	1	3.12E-10	NS	
PBMC	2889299	core	358328	2889241	LOC202134	5	rs10060053	0.456	Y	1	1.22E-11	0.01	
PBMC	2890160	extended	358859	2890148	HNRPH1	5	rs4128629	0.45	Y	1	3.79E-13	1.19E-07	
PBMC	2893430	full	360781	2893392	LY86	6	rs3789765	0.056	N	2	5.81E-10	NS	
PBMC	2897190	extended	363165	2897172	RNF144B	6	rs7748189	0.287	N	1	4.61E-12	0.003	
PBMC	2897185	core	363162	2897172	RNF144B	6	rs2328225	0.219	Y	2	2.33E-10	2.67E-08	
PBMC	2899153	core	364415	2899152	HIST1H2AC	6	rs1572982	0.494	Y	1	2.39E-15	7.82E-09	
PBMC	2899316	core	364502	2899298	BTN3A2	6	rs13195509	0.069	N	1	2.59E-10	0.018	
PBMC	2899374	core	364531	2899372	BTN3A1	6	rs10946829	0.156	Y	2	8.90E-12	NS	
PBMC	2899439	core	364570	2899437	BTN2A1	6	rs9295690	0.175	Y	1	2.05E-20	2.32E-06	
PBMC	2901279	full	365534	2901278	-	6	rs2517673	0.081	Y	1	7.76E-15	NS	
PBMC	2901297	ambiguous	365545	2901296	GENSCAN00000031134	6	rs9258966	0.297	Y	1	3.47E-14	0.001	
PBMC	2902222	extended	366014	2902221	-	6	rs2247056	0.269	Y	1	1.01E-12	5.35E-10	
PBMC	2903155	extended	366483	2903129	RNF5	6	rs2854050	0.044	Y	1	1.89E-10	NS	
PBMC	2903264	core	366547	2903258	HLA-DQA2	6	rs1063355	0.425	Y	1	2.71E-10	NS	
PBMC	2903266	core	366549	2903258	HLA-DQA2	6	rs10484561	0.1	Y	1	4.03E-10	NS	
PBMC	2903415	core	366630	2903401	HLA-DPB1	6	rs9277554	0.281	Y	1	2.55E-18	NS	
PBMC	2903423	core	366636	2903401	HLA-DPB1	6	rs3128917	0.231	Y	1	5.28E-14	5.63E-04	
PBMC	2903422	core	366635	2903401	HLA-DPB1	6	rs3128917	0.231	Y	2	3.65E-13	0.007	
PBMC	2905589	extended	367955	2905579	FLJ45825	6	rs1757171	0.356	Y	1	1.23E-17	NS	
PBMC	2905585	extended	367955	2905579	FLJ45825	6	rs1757171	0.356	N	1	8.01E-12	NS	
PBMC	2905591	extended	367957	2905579	FLJ45825	6	rs12211110	0.494	Y	1	4.96E-11	NS	
PBMC	2905588	extended	367955	2905579	FLJ45825	6	rs1757171	0.356	N	1	1.39E-10	NS	
PBMC	2905583	extended	367953	2905579	FLJ45825	6	rs1757171	0.356	N	1	2.50E-10	NS	
PBMC	2905602	extended	367964	2905600	-	6	rs1757171	0.356	Y	1	4.20E-15	NS	
PBMC	2905603	extended	367964	2905600	-	6	rs1757171	0.356	Y	1	2.24E-13	NS	
PBMC	2906643	extended	368660	2906607	NFYA	6	rs6905117	0.081	N	1	2.22E-14	NS	
PBMC	2906647	extended	368662	2906607	NFYA	6	rs6905117	0.081	N	1	1.63E-11	0.011	
PBMC	2925523	extended	380416	2925510	L3MBTL3	6	rs6569648	0.175	N	1	1.64E-13	NS	
PBMC	2925529	core	380422	2925510	L3MBTL3	6	rs7755589	0.234	Y	1	2.90E-11	3.93E-09	
PBMC	2946224	extended	393593	2946219	HIST1H2AB	6	rs1540275	0.294	Y	1	4.05E-11	NS	
PBMC	2947960	extended	394514	2947954	ZFP57	6	rs2747457	0.213	N	1	8.07E-24	NS	
PBMC	2947959	extended	394514	2947954	ZFP57	6	rs2535238	0.213	N	2	3.70E-16	NS	
PBMC	2947958	extended	394513	2947954	ZFP57	6	rs2747457	0.213	N	1	5.07E-12	NS	
PBMC	2948904	core	395044	2948887	HLA-C	6	rs6457374	0.269	Y	2	9.03E-16	8.54E-08	
PBMC	2948915	core	395049	2948887	HLA-C	6	rs6457374	0.269	Y	2	7.43E-14	6.45E-13	
PBMC	2948918	core	395051	2948887	HLA-C	6	rs2247056	0.269	Y	2	1.51E-12	2.06E-05	
PBMC	2948897	core	395039	2948887	HLA-C	6	rs6457374	0.269	Y	2	1.07E-11	2.28E-04	
PBMC	2948907	core	395044	2948887	HLA-C	6	rs2247056	0.269	Y	2	1.46E-10	2.11E-04	
PBMC	2948893	core	395037	2948887	HLA-C	6	rs9264942	0.344	Y	1	3.05E-10	3.62E-05	
PBMC	2948943	extended	395068	2948926	HLA-B	6	rs1058026	0.147	Y	2	5.49E-15	NS	
PBMC	2948948	extended	395069	2948926	HLA-B	6	rs2596503	0.181	Y	1	9.79E-13	NS	
PBMC	2948952	core	395071	2948926	HLA-B	6	rs2523554	0.35	Y	1	4.90E-10	0.022	
PBMC	2950128	core	395700	2950125	HLA-DQB2	6	rs1063355	0.425	Y	1	3.89E-13	NS	
PBMC	2950130	core	395702	2950125	HLA-DQB2	6	rs9275596	0.381	Y	1	5.56E-13	0.002	
PBMC	2950132	extended	395702	2950125	HLA-DQB2	6	rs3129727	0.05	Y	1	7.49E-11	NS	
PBMC	2950138	ambiguous	395707	2950137	-	6	rs1063355	0.425	Y	3	1.19E-20	4.81E-06	
PBMC	2950174	core	395724	2950167	TAP2	6	rs241448	0.226	Y	1	9.52E-12	5.99E-10	
PBMC	2950298	extended	395780	2950297	-	6	rs1480380	0.087	N	1	1.27E-12	5.02E-08	
PBMC	2950300	extended	395781	2950299	-	6	rs1480380	0.087	Y	1	1.64E-10	1.40E-04	
PBMC	2950304	extended	395783	2950303	AK055186	6	rs1480380	0.087	Y	1	6.00E-13	2.05E-04	
PBMC	2950343	core	395801	2950329	HLA-DPA1	6	rs2301220	0.194	Y	1	8.47E-12	7.14E-06	
PBMC	2950342	core	395801	2950329	HLA-DPA1	6	rs2301220	0.194	Y	1	1.68E-10	2.22E-06	
PBMC	2952353	core	397007	2952323	MDGA1	6	rs6938061	0.488	Y	1	3.71E-12	NS	
PBMC	2952358	core	397009	2952323	MDGA1	6	rs6938061	0.488	Y	1	7.94E-12	NS	
PBMC	2952340	core	396998	2952323	MDGA1	6	rs6938061	0.488	N	1	3.51E-11	NS	
PBMC	2952343	core	397001	2952323	MDGA1	6	rs6938061	0.488	N	1	3.61E-10	NS	
PBMC	2953164	core	397515	2953139	MOCS1	6	rs10046352	0.056	Y	1	1.26E-10	NS	
PBMC	2954286	core	398195	2954280	PEX6	6	rs6941212	0.419	Y	1	9.37E-14	6.13E-07	
PBMC	2963471	extended	403926	2963463	SNHG5	6	rs2842614	0.419	N	3	9.10E-14	0.001	
PBMC	2963467	extended	403926	2963463	SNHG5	6	rs2842614	0.419	N	3	1.56E-11	9.24E-04	
PBMC	2963473	extended	403927	2963463	SNHG5	6	rs2842614	0.419	N	1	2.52E-10	6.73E-04	
PBMC	2964472	extended	404566	2964350	MDN1	6	rs6913036	0.044	N	1	5.99E-10	NS	
PBMC	2965175	full	405021	2965174	-	6	rs9918469	0.05	Y	1	3.93E-10	NS	
PBMC	2968928	free	407381	2968953	MICAL1	6	rs7754650	0.406	Y	1	1.74E-10	NS	
PBMC	2971616	extended	409016	2971564	C6orf204	6	rs1343288	0.225	Y	1	2.92E-14	NS	
PBMC	2973450	full	410195	2973376	PTPRK	6	rs9398869	0.064	N	1	6.47E-11	NS	
PBMC	2974588	extended	410947	2974592	VNN1	6	rs2300077	0.338	N	1	3.02E-11	NS	
PBMC	2974600	core	410953	2974592	VNN1	6	rs2300077	0.338	Y	1	8.43E-11	NS	
PBMC	2974601	core	410954	2974592	VNN1	6	rs2300077	0.338	N	1	4.33E-10	NS	
PBMC	2975464	core	411507	2975385	AHI1	6	rs11154801	0.381	N	1	1.98E-10	0.036	
PBMC	2980494	full	414557	2980449	PIP3-E	6	rs1293927	0.325	N	1	9.55E-14	NS	
PBMC	2989124	core	419869	2989112	ZDHHC4	7	rs2346263	0.306	Y	1	6.67E-16	5.02E-09	
PBMC	2990060	extended	420460	2990043	PHF14	7	rs4720903	0.094	N	1	4.20E-10	NS	
PBMC	2996119	extended	424345	2996103	LOC441208	7	rs6950856	0.175	N	1	3.54E-11	NS	
PBMC	3003062	extended	428698	3003042	LOC442676	7	rs1113765	0.175	N	1	3.23E-14	3.05E-12	
PBMC	3003058	extended	428698	3003042	LOC442676	7	rs1113765	0.175	Y	3	1.24E-13	4.28E-08	
PBMC	3003064	extended	428698	3003042	LOC442676	7	rs1113765	0.175	Y	2	1.56E-13	5.00E-08	
PBMC	3003206	core	428790	3003193	CCT6A	7	rs4543497	0.272	Y	1	8.18E-13	1.18E-09	
PBMC	3018322	core	438087	3018309	PIK3CG	7	rs4730205	0.331	Y	1	9.84E-15	NS	
PBMC	3023264	core	441226	3023246	IRF5	7	rs6965542	0.412	Y	1	1.97E-12	3.61E-11	
PBMC	3023995	core	441685	3023964	CPA1	7	rs1544705	0.144	N	3	9.89E-11	NS	
PBMC	3028536	core	444578	3028217	TRBV19	7	rs2367486	0.424	Y	1	1.24E-17	0.008	
PBMC	3028509	core	444555	3028217	TRBV19	7	rs10231513	0.356	N	2	4.06E-11	NS	
PBMC	3028511	core	444556	3028217	TRBV19	7	rs10231513	0.356	N	2	2.05E-10	NS	
PBMC	3028661	ambiguous	444649	3028660	GENSCAN00000034084	7	rs2156940	0.287	Y	1	2.04E-10	NS	
PBMC	3031020	extended	446069	3030873	SSPO	7	rs6464035	0.344	N	3	6.83E-12	1.47E-05	
PBMC	3031636	core	446396	3031624	TMEM176A	7	rs7806458	0.356	Y	1	6.21E-26	NS	
PBMC	3031640	core	446396	3031624	TMEM176A	7	rs7806458	0.356	Y	1	1.83E-25	NS	
PBMC	3031646	core	446399	3031624	TMEM176A	7	rs7806458	0.356	Y	1	1.44E-24	NS	
PBMC	3031642	core	446397	3031624	TMEM176A	7	rs7806458	0.356	Y	2	5.08E-23	NS	
PBMC	3031638	core	446396	3031624	TMEM176A	7	rs7806458	0.356	Y	1	2.64E-18	NS	
PBMC	3031633	core	446394	3031624	TMEM176A	7	rs7806458	0.356	N	1	2.92E-11	NS	
PBMC	3036926	core	449662	3036924	ACTB	7	rs4320462	0.43	Y	1	2.59E-15	3.09E-06	
PBMC	3044217	extended	454197	3044152	LOC285949	7	rs38468	0.244	Y	1	9.13E-11	0.029	
PBMC	3044238	extended	454207	3044152	LOC285949	7	rs38494	0.213	Y	1	1.05E-10	1.94E-06	
PBMC	3044211	extended	454192	3044152	LOC285949	7	rs38494	0.213	N	1	4.21E-10	0.003	
PBMC	3044867	extended	454606	3044812	DPY19L1P1	7	rs6950856	0.175	N	2	3.89E-13	0.015	
PBMC	3044851	extended	454594	3044812	DPY19L1P1	7	rs6950856	0.175	N	2	4.46E-12	NS	
PBMC	3044843	extended	454586	3044812	DPY19L1P1	7	rs6950856	0.175	Y	2	1.81E-11	0.014	
PBMC	3048954	extended	457174	3048918	MYO1G	7	rs6976664	0.231	Y	1	6.25E-11	NS	
PBMC	3056323	core	461717	3056320	WBSCR27	7	rs4255023	0.291	N	1	1.25E-10	1.16E-04	
PBMC	3057764	core	462571	3057755	POMZP3	7	rs17718122	0.312	Y	3	6.69E-18	4.70E-10	
PBMC	3057758	core	462567	3057755	POMZP3	7	rs17718122	0.312	N	3	3.59E-14	1.01E-04	
PBMC	3067116	core	468396	3067080	COG5	7	rs2253833	0.287	Y	1	2.17E-10	NS	
PBMC	3067112	core	468394	3067080	COG5	7	rs2237659	0.294	Y	1	2.61E-10	0.039	
PBMC	3079182	core	476149	3079172	TMEM176B	7	rs7806458	0.356	Y	1	5.08E-24	NS	
PBMC	3079178	core	476146	3079172	TMEM176B	7	rs7806458	0.356	Y	1	1.18E-23	NS	
PBMC	3079180	core	476148	3079172	TMEM176B	7	rs7806458	0.356	Y	1	2.65E-19	0.032	
PBMC	3079174	core	476142	3079172	TMEM176B	7	rs7806458	0.356	Y	1	2.06E-17	NS	
PBMC	3079187	extended	476153	3079172	TMEM176B	7	rs7806458	0.356	Y	1	1.37E-16	NS	
PBMC	3079173	extended	476141	3079172	TMEM176B	7	rs7806458	0.356	N	1	2.61E-10	NS	
PBMC	3083068	core	478661	3082990	MYOM2	8	rs17831401	0.05	Y	1	1.90E-11	NS	
PBMC	3083063	core	478656	3082990	MYOM2	8	rs17831401	0.05	Y	1	3.04E-11	NS	
PBMC	3083071	core	478662	3082990	MYOM2	8	rs17831401	0.05	Y	1	3.07E-10	NS	
PBMC	3083053	extended	478646	3082990	MYOM2	8	rs17831401	0.05	Y	1	5.53E-10	NS	
PBMC	3083056	core	478649	3082990	MYOM2	8	rs9314454	0.144	N	1	6.13E-10	NS	
PBMC	3095821	full	486606	3095815	AGPAT6	8	rs6990606	0.469	Y	1	1.17E-11	0.022	
PBMC	3125381	core	505242	3125342	SGCZ	8	rs17120804	0.05	N	1	2.31E-10	NS	
PBMC	3125967	core	505623	3125915	MTUS1	8	rs17125630	0.07	N	2	1.94E-10	NS	
PBMC	3126063	extended	505689	3126062	-	8	rs4921823	0.05	Y	1	3.10E-10	NS	
PBMC	3130804	core	508616	3130757	FUT10	8	rs2348613	0.319	N	1	3.55E-11	0.017	
PBMC	3152763	extended	522481	3152752	-	8	rs10505473	0.169	N	1	9.63E-12	NS	
PBMC	3154151	core	523449	3154136	LRRC6	8	rs7011665	0.45	N	1	4.77E-10	NS	
PBMC	3154183	extended	523477	3154181	-	8	rs7011665	0.45	N	1	1.60E-10	NS	
PBMC	3157527	extended	525658	3157525	uc003yye.1	8	rs1077272	0.188	Y	2	1.11E-12	0.032	
PBMC	3158043	core	525939	3158011	PARP10	8	rs3936211	0.481	Y	1	2.52E-10	NS	
PBMC	3158518	core	526182	3158516	CPSF1	8	rs6599528	0.394	N	1	5.40E-10	NS	
PBMC	3158557	core	526206	3158516	CPSF1	8	rs3817681	0.375	N	2	5.74E-10	NS	
PBMC	3159373	full	526608	3159330	DOCK8	9	rs10970611	0.188	Y	3	8.09E-11	2.27E-04	
PBMC	3159374	full	526608	3159330	DOCK8	9	rs10970611	0.188	N	3	2.62E-10	1.53E-04	
PBMC	3160077	full	527059	3159946	SMARCA2	9	rs10738600	0.287	Y	1	2.63E-13	0.034	
PBMC	3160076	full	527059	3159946	SMARCA2	9	rs10738600	0.287	Y	1	4.15E-11	NS	
PBMC	3167394	core	531690	3167383	NUDT2	9	rs10972047	0.181	N	1	4.34E-10	NS	
PBMC	3168881	core	532593	3168841	GRHPR	9	rs10814535	0.346	N	1	2.17E-10	NS	
PBMC	3185029	extended	542311	3185028	-	9	rs7043162	0.481	Y	1	1.90E-12	4.42E-05	
PBMC	3187523	extended	543887	3187510	LOC253039	9	rs4837796	0.414	N	1	3.43E-25	5.55E-13	
PBMC	3187516	extended	543883	3187510	LOC253039	9	rs4837796	0.414	Y	1	1.80E-22	3.06E-14	
PBMC	3187525	extended	543887	3187510	LOC253039	9	rs4837796	0.414	Y	1	3.85E-22	6.59E-11	
PBMC	3187517	extended	543883	3187510	LOC253039	9	rs4837796	0.414	Y	1	2.48E-19	3.00E-11	
PBMC	3191608	extended	546462	3191589	FUBP3	9	rs7466269	0.375	Y	1	1.82E-10	5.57E-08	
PBMC	3195167	core	548576	3195139	UAP1L1	9	rs4880201	0.369	Y	1	2.01E-10	NS	
PBMC	3196566	full	549394	3196563	FLJ35024	9	rs11999258	0.05	N	1	1.86E-10	NS	
PBMC	3199665	extended	551438	3199662	C9orf52	9	rs7026350	0.237	Y	1	9.78E-20	3.93E-18	
PBMC	3200716	core	552109	3200689	RPS6	9	rs957	0.096	Y	2	3.22E-16	5.44E-13	
PBMC	3200692	extended	552090	3200689	RPS6	9	rs957	0.1	Y	2	4.15E-16	1.07E-11	
PBMC	3210510	extended	557967	3210497	PRUNE2	9	rs561970	0.218	Y	1	5.67E-13	NS	
PBMC	3210542	core	557990	3210497	PRUNE2	9	rs4744800	0.231	Y	1	1.75E-10	NS	
PBMC	3211972	extended	558924	3211938	RASEF	9	rs10867921	0.044	Y	1	2.51E-10	NS	
PBMC	3212038	extended	558963	3212008	FRMD3	9	rs11140081	0.044	Y	1	9.61E-11	NS	
PBMC	3212989	extended	559557	3212976	ZCCHC6	9	rs10115526	0.181	N	1	7.99E-12	2.86E-07	
PBMC	3220425	extended	564164	3220384	EDG2	9	rs1411424	0.381	Y	1	1.11E-11	NS	
PBMC	3220414	core	564155	3220384	EDG2	9	rs1411424	0.381	N	2	4.79E-11	NS	
PBMC	3220874	full	564443	3220846	SUSD1	9	rs13301075	0.045	N	1	1.07E-10	NS	
PBMC	3221949	core	565081	3221916	AKNA	9	rs1249726	0.494	Y	1	3.40E-14	NS	
PBMC	3229842	core	570069	3229837	CARD9	9	rs3812561	0.044	Y	1	8.82E-12	0.002	
PBMC	3230432	core	570376	3230414	LCN8	9	rs9886752	0.175	N	1	1.14E-10	NS	
PBMC	3238248	core	575248	3238231	MLLT10	10	rs11592565	0.369	Y	1	7.42E-17	8.93E-11	
PBMC	3240372	extended	576573	3240340	WAC	10	rs332144	0.488	Y	1	1.61E-13	2.74E-13	
PBMC	3252902	full	584421	3252690	C10orf11	10	rs10762726	0.152	N	1	1.09E-13	0.011	
PBMC	3265798	full	592506	3265734	GENSCAN00000067797	10	rs2530348	0.05	Y	1	2.20E-10	NS	
PBMC	3280698	full	601889	3280573	NEBL	10	rs7900067	0.125	N	1	3.89E-14	NS	
PBMC	3280628	full	601823	3280573	NEBL	10	rs7900067	0.125	N	1	2.63E-13	NS	
PBMC	3280627	full	601822	3280573	NEBL	10	rs10828213	0.112	N	1	4.17E-13	NS	
PBMC	3280625	full	601820	3280573	NEBL	10	rs7900067	0.125	N	1	2.24E-12	NS	
PBMC	3280580	full	601786	3280573	NEBL	10	rs7900067	0.125	N	2	2.30E-12	NS	
PBMC	3280614	full	601812	3280573	NEBL	10	rs7900067	0.125	Y	1	3.46E-12	NS	
PBMC	3280648	full	601841	3280573	NEBL	10	rs7900067	0.125	N	1	5.45E-12	NS	
PBMC	3280581	full	601787	3280573	NEBL	10	rs7900067	0.125	N	1	2.24E-11	NS	
PBMC	3280663	extended	601855	3280573	NEBL	10	rs3911716	0.125	Y	1	9.03E-11	NS	
PBMC	3280695	full	601887	3280573	NEBL	10	rs7900067	0.125	Y	1	2.42E-10	NS	
PBMC	3280576	core	601785	3280573	NEBL	10	rs7900067	0.125	Y	1	2.54E-10	NS	
PBMC	3280624	full	601819	3280573	NEBL	10	rs7900067	0.125	N	1	4.47E-10	NS	
PBMC	3280693	full	601885	3280573	NEBL	10	rs7900067	0.125	N	1	5.22E-10	NS	
PBMC	3293471	full	609981	3293469	C10orf27	10	rs7092269	0.044	N	1	4.51E-10	NS	
PBMC	3293800	core	610178	3293762	PSAP	10	rs7905774	0.106	N	2	3.69E-13	1.58E-05	
PBMC	3299599	core	613768	3299585	LIPA	10	rs2243547	0.325	N	2	4.68E-14	NS	
PBMC	3299600	core	613768	3299585	LIPA	10	rs2243547	0.325	N	1	5.89E-11	NS	
PBMC	3299590	core	613762	3299585	LIPA	10	rs1412444	0.35	N	1	1.00E-10	NS	
PBMC	3302380	core	615535	3302360	MMS19	10	rs3740526	0.425	Y	1	2.43E-10	0.007	
PBMC	3302379	core	615534	3302360	MMS19	10	rs3740526	0.425	N	1	4.07E-10	0.004	
PBMC	3303657	core	616331	3303652	MRPL43	10	rs2863095	0.231	N	1	3.44E-11	0.01	
PBMC	3303658	core	616331	3303652	MRPL43	10	rs2863095	0.231	N	1	1.08E-10	1.21E-06	
PBMC	3308526	core	619403	3308489	KIAA1598	10	rs10787735	0.275	N	1	4.14E-11	NS	
PBMC	3308493	core	619380	3308489	KIAA1598	10	rs10787735	0.275	N	1	5.07E-11	NS	
PBMC	3308517	core	619395	3308489	KIAA1598	10	rs740600	0.269	N	1	1.02E-10	NS	
PBMC	3308546	core	619419	3308489	KIAA1598	10	rs2257791	0.237	N	1	4.58E-10	NS	
PBMC	3311496	core	621257	3311417	CTBP2	10	rs17152617	0.194	Y	3	8.55E-11	1.23E-08	
PBMC	3312145	full	621697	3312045	C10orf90	10	rs17691586	0.044	Y	2	3.79E-12	NS	
PBMC	3315522	core	623781	3315512	RIC8A	11	rs1045454	0.244	Y	1	1.33E-11	NS	
PBMC	3315532	core	623787	3315512	RIC8A	11	rs7128029	0.253	N	1	6.50E-11	NS	
PBMC	3315520	core	623779	3315512	RIC8A	11	rs1045454	0.244	Y	1	1.51E-10	NS	
PBMC	3315697	extended	623868	3315696	-	11	rs7395319	0.138	N	3	4.14E-10	NS	
PBMC	3319298	core	625961	3319287	EIF3F	11	rs7951923	0.169	Y	3	9.32E-19	1.03E-08	
PBMC	3328369	core	631736	3328349	PHACS	11	rs7127924	0.194	Y	1	9.93E-12	0.001	
PBMC	3328359	core	631733	3328349	PHACS	11	rs7951555	0.188	Y	1	2.32E-11	NS	
PBMC	3328363	core	631736	3328349	PHACS	11	rs7127924	0.194	N	1	3.33E-11	NS	
PBMC	3328380	core	631742	3328349	PHACS	11	rs7127924	0.194	Y	1	1.87E-10	0.025	
PBMC	3332338	extended	633991	3332334	NYD-SP21	11	rs10792269	0.325	N	1	8.27E-11	NS	
PBMC	3334570	core	635297	3334518	CCDC88B	11	rs538147	0.387	Y	2	3.63E-11	NS	
PBMC	3337192	core	636728	3337168	GSTP1	11	rs1695	0.287	Y	1	5.58E-16	4.47E-07	
PBMC	3337957	core	637197	3337918	TPCN2	11	rs1005858	0.45	Y	2	4.68E-23	3.90E-08	
PBMC	3337990	extended	637216	3337918	TPCN2	11	rs1060435	0.45	N	2	5.98E-12	9.49E-07	
PBMC	3338995	extended	637813	3338968	NADSYN1	11	rs7940244	0.213	Y	1	2.55E-11	5.03E-04	
PBMC	3338978	extended	637802	3338968	NADSYN1	11	rs12800438	0.244	Y	1	4.55E-11	NS	
PBMC	3339361	core	638008	3339346	FOLR3	11	rs7925545	0.075	N	3	6.75E-14	NS	
PBMC	3339357	core	638007	3339346	FOLR3	11	rs7925545	0.075	N	1	1.04E-11	NS	
PBMC	3339365	core	638009	3339346	FOLR3	11	rs7925545	0.075	N	1	3.08E-10	NS	
PBMC	3339384	full	638021	3339382	FOLR2	11	rs7925545	0.075	N	3	2.95E-11	NS	
PBMC	3339401	full	638032	3339399	FOLR3	11	rs7925545	0.075	N	3	1.26E-13	NS	
PBMC	3339402	extended	638033	3339399	FOLR3	11	rs7925545	0.075	N	3	5.02E-13	NS	
PBMC	3344708	core	641257	3344685	CCDC67	11	rs1579874	0.069	N	1	1.41E-10	NS	
PBMC	3369259	core	656361	3369249	APIP	11	rs1326944	0.225	Y	1	1.04E-12	4.35E-06	
PBMC	3398516	extended	673941	3398482	SNX19	11	rs12418278	0.25	N	1	5.59E-11	3.61E-05	
PBMC	3402541	core	676555	3402522	TAPBPL	12	rs2041385	0.256	Y	1	4.21E-10	0.003	
PBMC	3403368	extended	677040	3403367	-	12	rs7487128	0.044	N	1	3.17E-11	NS	
PBMC	3407806	extended	679806	3407793	PYROXD1	12	rs738027	0.363	Y	1	1.44E-10	NS	
PBMC	3407830	extended	679822	3407824	GOLT1B	12	rs17681644	0.069	N	2	4.32E-10	NS	
PBMC	3410085	core	681272	3410060	DDX11	12	rs10843881	0.475	Y	3	3.17E-12	NS	
PBMC	3410094	core	681278	3410060	DDX11	12	rs10843881	0.475	Y	3	1.49E-11	1.14E-04	
PBMC	3410111	core	681289	3410060	DDX11	12	rs10843881	0.475	N	3	1.88E-11	4.59E-05	
PBMC	3410115	extended	681290	3410060	DDX11	12	rs10843881	0.475	Y	1	6.03E-10	0.007	
PBMC	3413138	extended	683232	3413067	FAM113B	12	rs712099	0.35	Y	1	3.11E-13	3.02E-04	
PBMC	3416031	core	684868	3416019	PRR13	12	rs2683525	0.162	Y	3	1.04E-10	1.99E-05	
PBMC	3417242	core	685528	3417240	RPS26	12	rs10876864	0.412	N	2	1.24E-38	2.23E-14	
PBMC	3417244	core	685528	3417240	RPS26	12	rs10876864	0.412	N	2	6.92E-37	7.07E-21	
PBMC	3417247	core	685531	3417240	RPS26	12	rs10876864	0.412	N	2	3.56E-24	1.14E-06	
PBMC	3417246	core	685530	3417240	RPS26	12	rs10876864	0.412	N	2	5.58E-19	0.031	
PBMC	3423261	full	689152	3423260	-	12	rs12371787	0.044	N	1	5.12E-11	NS	
PBMC	3424096	extended	689721	3424076	ENST00000266688	12	rs1528287	0.044	N	1	1.61E-10	NS	
PBMC	3426230	extended	691040	3426215	MRPL42	12	rs11107052	0.167	Y	1	3.70E-14	4.72E-12	
PBMC	3426524	full	691229	3426502	PLXNC1	12	rs3847804	0.206	Y	1	1.11E-14	NS	
PBMC	3432458	core	695063	3432438	OAS1	12	rs3177979	0.375	N	1	2.84E-10	0.002	
PBMC	3444388	extended	702303	3444368	PRH1	12	rs7486717	0.463	Y	1	1.53E-13	4.66E-04	
PBMC	3444529	extended	702374	3444528	-	12	rs6488331	0.156	Y	1	9.98E-15	4.80E-13	
PBMC	3449120	core	705225	3449068	TMTC1	12	rs4931215	0.431	N	1	5.43E-11	NS	
PBMC	3449638	extended	705557	3449591	OVOS2	12	rs11051351	0.275	N	2	5.44E-10	NS	
PBMC	3451194	full	706543	3451193	ENST00000387167	12	rs17591910	0.044	N	2	6.76E-11	NS	
PBMC	3451943	full	707001	3451942	DBX2	12	rs11182800	0.05	N	2	1.17E-10	NS	
PBMC	3453692	core	708035	3453592	MLL2	12	rs12580349	0.331	Y	2	3.50E-14	1.10E-04	
PBMC	3458086	extended	710516	3458057	PTGES3	12	rs3214051	0.394	Y	1	3.19E-11	5.19E-04	
PBMC	3458101	core	710523	3458097	NACA	12	rs1465081	0.3	Y	2	1.00E-12	1.52E-08	
PBMC	3462877	core	713445	3462843	NAP1L1	12	rs2043369	0.369	Y	3	3.12E-29	8.47E-14	
PBMC	3464448	extended	714441	3464417	MGAT4C	12	rs12311449	0.044	Y	1	1.24E-11	NS	
PBMC	3472315	core	719427	3472312	SLC24A6	12	rs2303620	0.087	Y	1	3.17E-13	0.004	
PBMC	3475301	core	721367	3475295	MORN3	12	rs12320939	0.35	N	2	5.56E-12	NS	
PBMC	3478470	core	723383	3478457	STX2	12	rs10773819	0.419	N	1	1.53E-10	NS	
PBMC	3479368	core	723912	3479355	GOLGA3	12	rs7136989	0.081	Y	1	6.96E-16	4.91E-07	
PBMC	3485880	core	727948	3485863	EXOSC8	13	rs9469	0.4	Y	1	1.62E-15	4.78E-13	
PBMC	3486060	core	728049	3486025	UFM1	13	rs6563610	0.054	Y	1	8.72E-13	5.65E-12	
PBMC	3487448	core	728937	3487432	DNAJC15	13	rs7325480	0.441	Y	1	7.22E-17	8.71E-12	
PBMC	3502864	full	738698	3502829	GAS6	13	rs9604573	0.3	Y	3	4.71E-16	6.99E-04	
PBMC	3509914	core	743058	3509910	FAM48A	13	rs2670061	0.481	Y	1	1.79E-10	NS	
PBMC	3524925	extended	752430	3524924	-	13	rs16971270	0.044	N	1	1.88E-10	NS	
PBMC	3527423	core	753898	3527418	PARP2	14	rs3742945	0.219	N	1	5.06E-11	4.73E-09	
PBMC	3535924	core	758969	3535922	STYX	14	rs12589690	0.381	Y	1	2.65E-11	2.48E-05	
PBMC	3540371	core	761793	3540353	CHURC1	14	rs7143432	0.225	Y	1	3.34E-10	NS	
PBMC	3547553	core	766368	3547500	SPATA7	14	rs12433026	0.369	N	1	7.50E-11	1.07E-04	
PBMC	3555408	core	771220	3555340	TEP1	14	rs4982038	0.297	Y	1	2.30E-12	1.70E-05	
PBMC	3557566	extended	772377	3557504	MYH7	14	rs2295705	0.044	N	1	7.30E-11	NS	
PBMC	3558176	extended	772666	3558168	ADCY4	14	rs10129213	0.05	N	2	4.51E-10	NS	
PBMC	3558301	extended	772714	3558290	C14orf124	14	rs11625819	0.106	Y	1	3.21E-10	7.04E-07	
PBMC	3568521	extended	779026	3568534	SPTB	14	rs7143124	0.044	N	1	1.01E-10	NS	
PBMC	3586789	ambiguous	790343	3586788	-	15	rs1524878	0.475	N	3	1.86E-10	NS	
PBMC	3590367	extended	792480	3590365	LOC729082	15	rs7178777	0.381	Y	1	2.50E-11	2.90E-09	
PBMC	3595463	core	795653	3595441	Gcom1	15	rs4774966	0.481	Y	1	5.66E-11	NS	
PBMC	3595452	core	795645	3595441	Gcom1	15	rs4774966	0.481	N	1	1.79E-10	NS	
PBMC	3595464	core	795654	3595441	Gcom1	15	rs11632793	0.294	N	1	2.16E-10	NS	
PBMC	3595459	core	795651	3595441	Gcom1	15	rs11632793	0.294	N	1	3.93E-10	NS	
PBMC	3596497	full	796286	3596491	GENSCAN00000044496	15	rs1680446	0.044	N	2	9.07E-12	NS	
PBMC	3597185	full	796693	3597125	TLN2	15	rs8033596	0.044	N	1	1.36E-11	NS	
PBMC	3616958	extended	808920	3616796	FMN1	15	rs345776	0.194	N	1	1.28E-15	NS	
PBMC	3616801	extended	808807	3616796	FMN1	15	rs345776	0.194	N	1	1.59E-14	NS	
PBMC	3616904	extended	808893	3616796	FMN1	15	rs345776	0.194	Y	1	2.26E-13	NS	
PBMC	3616804	extended	808808	3616796	FMN1	15	rs345776	0.194	N	1	4.00E-13	NS	
PBMC	3616910	extended	808897	3616796	FMN1	15	rs345776	0.194	Y	1	2.14E-12	NS	
PBMC	3616808	extended	808810	3616796	FMN1	15	rs345776	0.194	Y	1	2.94E-12	NS	
PBMC	3616956	extended	808920	3616796	FMN1	15	rs345776	0.194	N	1	6.57E-12	NS	
PBMC	3616962	extended	808923	3616796	FMN1	15	rs345776	0.194	N	1	1.03E-11	NS	
PBMC	3616984	extended	808934	3616796	FMN1	15	rs12910096	0.1	N	1	1.17E-10	NS	
PBMC	3616946	extended	808915	3616796	FMN1	15	rs12910096	0.1	N	1	5.04E-10	NS	
PBMC	3616828	extended	808829	3616796	FMN1	15	rs345776	0.194	N	1	5.67E-10	NS	
PBMC	3617517	extended	809265	3617458	GOLGA8A	15	rs4924045	0.12	Y	2	2.18E-18	4.91E-04	
PBMC	3617521	extended	809269	3617458	GOLGA8A	15	rs8041207	0.35	Y	1	1.57E-14	0.006	
PBMC	3617640	ambiguous	809336	3617639	GOLGA8B	15	rs8041207	0.35	Y	1	4.65E-12	2.88E-06	
PBMC	3620695	extended	811251	3620683	LRRC57	15	rs4924687	0.125	Y	1	5.09E-17	7.75E-17	
PBMC	3626696	extended	815057	3626689	-	15	rs650731	0.05	Y	1	3.44E-11	NS	
PBMC	3629253	core	816721	3629243	RBPMS2	15	rs7174486	0.05	N	1	1.18E-11	NS	
PBMC	3629251	core	816720	3629243	RBPMS2	15	rs7174486	0.05	N	1	5.86E-11	NS	
PBMC	3629252	core	816720	3629243	RBPMS2	15	rs7174486	0.05	N	1	2.61E-10	0.007	
PBMC	3633368	extended	819208	3633347	MAN2C1	15	rs8028182	0.219	Y	1	1.85E-17	2.19E-05	
PBMC	3633397	core	819224	3633347	MAN2C1	15	rs4886699	0.256	Y	1	1.71E-11	NS	
PBMC	3633352	core	819200	3633347	MAN2C1	15	rs4886699	0.256	Y	1	1.88E-11	NS	
PBMC	3633375	core	819212	3633347	MAN2C1	15	rs4886699	0.256	N	1	5.31E-11	0.039	
PBMC	3633380	core	819216	3633347	MAN2C1	15	rs8028182	0.219	N	1	1.11E-10	NS	
PBMC	3633381	core	819217	3633347	MAN2C1	15	rs4886699	0.256	N	1	2.24E-10	NS	
PBMC	3633364	core	819208	3633347	MAN2C1	15	rs4886699	0.256	N	1	4.79E-10	NS	
PBMC	3635185	extended	820384	3635184	AK094053	15	rs6495456	0.331	Y	1	3.84E-13	8.44E-10	
PBMC	3638706	core	822512	3638699	C15orf38	15	rs1256854	0.2	N	1	1.64E-11	NS	
PBMC	3638704	core	822510	3638699	C15orf38	15	rs1256854	0.2	Y	1	3.27E-10	NS	
PBMC	3642128	extended	824666	3642127	-	15	rs1975752	0.425	Y	1	1.79E-11	1.13E-08	
PBMC	3647874	core	828041	3647827	ATF7IP2	16	rs11645643	0.412	N	1	1.75E-10	9.64E-06	
PBMC	3654875	extended	832280	3654859	ATXN2L	16	rs4788102	0.338	Y	2	1.24E-11	0.01	
PBMC	3661088	extended	835921	3661065	RBL2	16	rs8043918	0.463	Y	1	6.67E-19	0.001	
PBMC	3674128	extended	843932	3674048	SPG7	16	rs17775174	0.188	Y	1	1.45E-17	7.31E-12	
PBMC	3675138	core	844508	3675116	TMEM8	16	rs2071915	0.449	Y	1	1.61E-14	NS	
PBMC	3675137	core	844507	3675116	TMEM8	16	rs3830160	0.406	N	1	3.57E-10	0.025	
PBMC	3676729	free	845410	3676669	RNPS1	16	rs155243	0.394	Y	1	5.17E-11	8.28E-08	
PBMC	3680501	full	847654	3680479	TXNDC11	16	rs8191288	0.419	Y	1	3.03E-14	2.27E-08	
PBMC	3686090	core	850957	3686080	NSMCE1	16	rs7195219	0.363	Y	1	1.36E-10	6.15E-06	
PBMC	3686740	core	851353	3686728	TUFM	16	rs4788102	0.338	N	2	1.52E-10	NS	
PBMC	3687051	extended	851518	3686992	BOLA2	16	rs11859842	0.494	Y	1	3.93E-10	3.02E-07	
PBMC	3687655	extended	851847	3687632	LOC641298	16	rs11862806	0.312	N	2	5.13E-11	3.18E-06	
PBMC	3702266	full	860796	3702262	LOC146167	16	rs7201492	0.05	Y	1	1.40E-12	NS	
PBMC	3715277	core	868579	3715274	LGALS9	17	rs4794975	0.25	Y	2	1.88E-10	NS	
PBMC	3723579	extended	873432	3723572	C17orf69	17	rs393152	0.269	Y	1	4.97E-13	3.53E-05	
PBMC	3723575	extended	873428	3723572	C17orf69	17	rs7215239	0.306	Y	3	6.02E-10	4.91E-06	
PBMC	3723867	extended	873600	3723866	-	17	rs2532269	0.211	Y	1	1.15E-15	2.65E-13	
PBMC	3723937	core	873631	3723891	LRRC37A	17	rs2532329	0.191	N	3	3.15E-13	4.96E-13	
PBMC	3726599	core	875228	3726569	SPATA20	17	rs989128	0.325	Y	1	6.30E-15	4.26E-05	
PBMC	3726605	core	875232	3726569	SPATA20	17	rs989128	0.325	N	1	1.29E-11	0.002	
PBMC	3733108	extended	879279	3733065	MAP2K6	17	rs2715833	0.363	Y	1	8.97E-12	8.83E-06	
PBMC	3735020	extended	880427	3734966	MYO15B	17	rs4788901	0.062	Y	1	4.23E-20	NS	
PBMC	3735035	extended	880439	3734966	MYO15B	17	rs4788901	0.062	N	1	1.05E-18	NS	
PBMC	3735033	extended	880437	3734966	MYO15B	17	rs4788901	0.062	N	1	1.99E-15	NS	
PBMC	3735002	extended	880415	3734966	MYO15B	17	rs4788901	0.062	N	1	2.34E-14	NS	
PBMC	3735048	extended	880447	3734966	MYO15B	17	rs4788901	0.062	N	1	1.98E-13	NS	
PBMC	3735004	extended	880416	3734966	MYO15B	17	rs4788901	0.062	N	1	2.61E-13	NS	
PBMC	3735036	extended	880440	3734966	MYO15B	17	rs4788901	0.062	N	1	2.84E-13	NS	
PBMC	3735026	extended	880432	3734966	MYO15B	17	rs4788901	0.062	Y	1	5.56E-13	NS	
PBMC	3735017	extended	880424	3734966	MYO15B	17	rs4788901	0.062	N	1	8.90E-13	NS	
PBMC	3735024	extended	880430	3734966	MYO15B	17	rs4788901	0.062	N	1	4.72E-12	NS	
PBMC	3735081	core	880471	3734966	MYO15B	17	rs4788901	0.062	N	1	1.39E-11	NS	
PBMC	3735025	extended	880431	3734966	MYO15B	17	rs4788901	0.062	N	1	2.69E-11	NS	
PBMC	3735068	core	880461	3734966	MYO15B	17	rs4788901	0.062	N	1	9.76E-11	NS	
PBMC	3735014	extended	880423	3734966	MYO15B	17	rs4788901	0.062	N	1	1.14E-10	NS	
PBMC	3735049	extended	880448	3734966	MYO15B	17	rs4788901	0.062	Y	1	1.20E-10	NS	
PBMC	3735066	core	880459	3734966	MYO15B	17	rs4788901	0.062	N	1	1.79E-10	NS	
PBMC	3735059	extended	880456	3734966	MYO15B	17	rs4788901	0.062	N	1	2.03E-10	NS	
PBMC	3734984	extended	880408	3734966	MYO15B	17	rs4788901	0.062	Y	1	2.69E-10	NS	
PBMC	3735061	extended	880457	3734966	MYO15B	17	rs4788901	0.062	N	1	3.90E-10	NS	
PBMC	3735080	core	880470	3734966	MYO15B	17	rs2290454	0.062	N	1	4.51E-10	0.003	
PBMC	3735001	extended	880415	3734966	MYO15B	17	rs4788901	0.062	Y	1	5.07E-10	NS	
PBMC	3735754	extended	880854	3735752	SEC14L1	17	rs716284	0.194	Y	1	2.24E-12	2.19E-08	
PBMC	3737315	extended	881832	3737274	KIAA1618	17	rs10782008	0.375	Y	1	5.94E-10	9.24E-09	
PBMC	3741835	core	884539	3741800	ATP2A3	17	rs758641	0.431	Y	1	3.92E-20	NS	
PBMC	3755366	extended	892568	3755359	PIP4K2B	17	rs228249	0.281	Y	1	2.39E-10	9.78E-05	
PBMC	3757681	extended	893875	3757664	RAB5C	17	rs1122326	0.294	Y	1	3.12E-10	0.011	
PBMC	3758443	extended	894323	3758430	ENST00000341011	17	rs11651341	0.319	Y	1	7.41E-11	2.20E-05	
PBMC	3759895	extended	895198	3759894	-	17	rs11012	0.219	Y	1	5.15E-10	0.013	
PBMC	3759898	extended	895200	3759897	LRRC37A4	17	rs11012	0.219	Y	1	5.73E-15	2.70E-04	
PBMC	3759900	extended	895201	3759897	LRRC37A4	17	rs11012	0.219	N	2	1.64E-11	2.42E-06	
PBMC	3760017	extended	895262	3760013	BC019018	17	rs393152	0.269	N	2	1.11E-12	1.81E-08	
PBMC	3760024	extended	895264	3760013	BC019018	17	rs393152	0.269	N	2	2.25E-10	8.02E-06	
PBMC	3760019	extended	895262	3760013	BC019018	17	rs417968	0.325	N	3	5.27E-10	NS	
PBMC	3760199	extended	895386	3760137	KIAA1267	17	rs2532269	0.211	N	1	4.47E-18	2.39E-16	
PBMC	3760146	core	895344	3760137	KIAA1267	17	rs1981997	0.275	Y	1	2.49E-10	7.79E-06	
PBMC	3764386	core	897914	3764384	SUPT4H1	17	rs3744093	0.394	Y	1	1.19E-12	3.92E-12	
PBMC	3770322	full	901644	3770321	LOC441800	17	rs8076892	0.131	N	1	5.26E-11	NS	
PBMC	3774549	core	904073	3774535	DCXR	17	rs4969481	0.144	N	1	1.69E-10	NS	
PBMC	3775179	core	904426	3775157	WDR45L	17	rs3826310	0.231	Y	2	7.75E-12	3.19E-07	
PBMC	3776450	extended	905219	3776449	MRLC2	18	rs717183	0.344	Y	1	2.60E-16	2.16E-06	
PBMC	3778839	extended	906734	3778823	NAPG	18	rs546966	0.325	Y	1	1.02E-10	2.72E-06	
PBMC	3779595	extended	907197	3779579	TUBB6	18	rs11080567	0.138	Y	1	9.99E-12	NS	
PBMC	3814950	extended	929681	3814937	BC010544	19	rs2238585	0.275	N	1	2.16E-11	NS	
PBMC	3818463	extended	931715	3818462	-	19	rs8111177	0.425	Y	1	4.35E-14	NS	
PBMC	3821688	extended	933536	3821603	ZNF791	19	rs983149	0.325	Y	1	8.51E-11	NS	
PBMC	3832977	extended	940139	3832964	MED29	19	rs10425594	0.356	Y	1	9.66E-14	0.027	
PBMC	3841098	core	944686	3841076	MYADM	19	rs8103771	0.062	Y	1	2.02E-11	NS	
PBMC	3841844	core	945121	3841838	KIR2DS4	19	rs11665986	0.188	Y	2	6.25E-12	NS	
PBMC	3847913	core	948531	3847906	DENND1C	19	rs8111177	0.425	Y	1	1.26E-14	NS	
PBMC	3849691	core	949584	3849688	ZNF266	19	rs10401135	0.431	N	2	4.20E-12	4.51E-04	
PBMC	3849700	core	949588	3849688	ZNF266	19	rs10401135	0.431	N	2	3.12E-10	0.024	
PBMC	3856515	extended	953438	3856513	-	19	rs1401926	0.463	Y	1	4.08E-13	1.03E-06	
PBMC	3856517	extended	953439	3856516	-	19	rs1401926	0.463	Y	1	1.44E-10	2.54E-08	
PBMC	3859902	core	955509	3859899	TMEM149	19	rs8106959	0.15	N	1	3.81E-10	0.004	
PBMC	3869796	ambiguous	961053	3869795	ZNF28	19	rs1062967	0.468	N	1	7.40E-11	3.71E-06	
PBMC	3870671	core	961517	3870611	LILRB3	19	rs103294	0.181	Y	2	4.63E-11	NS	
PBMC	3870663	core	961513	3870611	LILRB3	19	rs103294	0.181	N	2	5.85E-11	NS	
PBMC	3870667	core	961515	3870611	LILRB3	19	rs103294	0.181	N	1	1.09E-10	NS	
PBMC	3870670	core	961517	3870611	LILRB3	19	rs103294	0.181	N	1	2.12E-10	NS	
PBMC	3870674	core	961518	3870611	LILRB3	19	rs103294	0.181	N	1	4.80E-10	NS	
PBMC	3870745	core	961549	3870733	LILRB2	19	rs6509860	0.369	Y	2	2.62E-12	NS	
PBMC	3873734	extended	963182	3873725	LOC388780	20	rs6113074	0.062	Y	1	2.89E-12	NS	
PBMC	3880161	extended	967221	3880154	AK091765	20	rs6137866	0.156	N	1	4.19E-10	NS	
PBMC	3894743	extended	976105	3894727	SIRPB1	20	rs11696842	0.262	N	1	2.19E-30	0.007	
PBMC	3894757	core	976114	3894727	SIRPB1	20	rs1535882	0.399	Y	2	4.74E-11	NS	
PBMC	3904125	core	981737	3904119	CPNE1	20	rs11696527	0.087	N	1	2.39E-10	0.001	
PBMC	3909730	extended	985214	3909642	ATP9A	20	rs7261003	0.463	N	1	2.41E-10	NS	
PBMC	3914253	full	987942	3914230	ZNF512B	20	rs817329	0.456	N	2	1.67E-10	2.06E-08	
PBMC	3914266	extended	987950	3914264	-	20	rs817329	0.456	N	2	1.06E-14	6.64E-14	
PBMC	3914265	extended	987949	3914264	-	20	rs817329	0.456	Y	2	1.85E-11	1.90E-11	
PBMC	3917254	full	989864	3917204	C21orf7	21	rs2832279	0.35	N	1	1.58E-11	NS	
PBMC	3922940	extended	993363	3922921	NDUFV3	21	rs2839603	0.087	N	1	1.10E-20	1.08E-04	
PBMC	3924803	core	994556	3924783	PRMT2	21	rs15047	0.131	Y	1	6.74E-11	4.77E-09	
PBMC	3926119	full	995351	3926080	BTG3	21	rs2246741	0.044	N	1	2.97E-11	0.023	
PBMC	3934759	extended	1000622	3934729	ITGB2	21	rs760462	0.169	Y	1	7.29E-22	NS	
PBMC	3938418	extended	1002689	3938384	IGL@	22	rs3819309	0.181	N	2	1.73E-10	1.78E-05	
PBMC	3946810	core	1007612	3946762	ZC3H7B	22	rs2024566	0.356	N	1	5.63E-10	0.012	
PBMC	3947316	core	1007911	3947310	C22orf32	22	rs1801311	0.325	Y	1	2.58E-16	0.004	
PBMC	3947312	core	1007909	3947310	C22orf32	22	rs1801311	0.325	N	1	2.40E-11	0.011	
PBMC	3948487	core	1008658	3948461	NUP50	22	rs13058199	0.3	N	2	1.98E-11	1.62E-09	
PBMC	3948569	extended	1008699	3948543	FAM118A	22	rs104664	0.175	N	1	3.11E-21	2.06E-09	
PBMC	3948568	extended	1008698	3948543	FAM118A	22	rs104664	0.175	N	1	1.88E-16	5.89E-13	
PBMC	3948570	core	1008699	3948543	FAM118A	22	rs104664	0.175	Y	1	4.01E-16	2.25E-05	
PBMC	3948566	extended	1008697	3948543	FAM118A	22	rs104664	0.175	Y	1	5.47E-15	1.33E-10	
PBMC	3948567	extended	1008698	3948543	FAM118A	22	rs104664	0.175	Y	1	2.84E-14	5.37E-11	
PBMC	3948556	core	1008694	3948543	FAM118A	22	rs104664	0.175	Y	1	5.91E-13	NS	
PBMC	3948557	core	1008694	3948543	FAM118A	22	rs6007594	0.3	Y	3	1.54E-12	1.39E-06	
PBMC	3948572	core	1008700	3948543	FAM118A	22	rs104664	0.175	Y	1	9.59E-12	2.02E-05	
PBMC	3948577	core	1008703	3948543	FAM118A	22	rs104664	0.175	N	1	1.50E-10	2.89E-04	
PBMC	3948579	core	1008704	3948543	FAM118A	22	rs104664	0.175	N	2	3.03E-10	3.18E-06	
PBMC	3954834	extended	1012483	3954764	IGLL3	22	rs6003847	0.188	N	2	7.39E-12	6.13E-06	
PBMC	3954988	extended	1012555	3954987	-	22	rs5760102	0.363	N	1	1.10E-11	NS	
PBMC	3958442	core	1014603	3958422	BPIL2	22	rs1109363	0.081	N	1	8.98E-11	8.18E-04	
PBMC	3965108	extended	1018715	3965102	C22orf34	22	rs9616329	0.231	N	1	7.79E-11	NS	
PBMC	3965103	extended	1018713	3965102	C22orf34	22	rs2071904	0.344	Y	1	5.17E-10	NS	
PBMC	3965130	extended	1018735	3965102	C22orf34	22	rs9616329	0.231	Y	1	5.81E-10	NS	
PBMC	4048240	extended	1068860	4048230	GENSCAN00000036911	6	rs9271366	0.169	Y	1	5.23E-16	NS	
PBMC	4048242	core	1068861	4048241	HLA-DRB5	6	rs9271366	0.169	N	2	1.92E-26	0.006	
PBMC	4048252	core	1068868	4048241	HLA-DRB5	6	rs9271366	0.169	Y	1	2.16E-25	5.69E-07	
PBMC	4048243	core	1068862	4048241	HLA-DRB5	6	rs9271366	0.169	Y	1	1.03E-20	1.56E-07	
PBMC	4048247	core	1068866	4048241	HLA-DRB5	6	rs9271366	0.169	Y	2	1.55E-20	5.54E-05	
PBMC	4048253	core	1068868	4048241	HLA-DRB5	6	rs9271366	0.169	Y	2	4.80E-16	5.29E-08	
PBMC	4048248	core	1068866	4048241	HLA-DRB5	6	rs3129860	0.181	N	2	2.64E-10	0.045	
PBMC	4048258	extended	1068872	4048241	HLA-DRB5	6	rs3129860	0.181	Y	3	5.12E-10	0.042	
PBMC	4048293	extended	1068892	4048265	HLA-DRB1	6	rs9271366	0.169	Y	1	5.12E-20	NS	
PBMC	4048290	core	1068890	4048265	HLA-DRB1	6	rs9271366	0.169	Y	1	1.86E-18	2.79E-10	
PBMC	4048279	core	1068882	4048265	HLA-DRB1	6	rs9271366	0.169	Y	1	3.84E-18	7.07E-10	
PBMC	4048291	full	1068890	4048265	HLA-DRB1	6	rs9271366	0.169	Y	2	3.52E-15	NS	
PBMC	4048277	full	1068880	4048265	HLA-DRB1	6	rs2858870	0.175	Y	2	1.96E-14	2.33E-05	
PBMC	4048286	full	1068888	4048265	HLA-DRB1	6	rs9271366	0.169	Y	1	4.59E-14	NS	
PBMC	4048289	core	1068890	4048265	HLA-DRB1	6	rs9271366	0.169	Y	1	2.67E-13	1.13E-07	
PBMC	4048285	core	1068887	4048265	HLA-DRB1	6	rs9271366	0.169	Y	2	7.28E-13	1.55E-06	
a	Level is defined by Affymetrix, depending on the level of literature support for the exon, with confidence hierarchy core>extended>full.				
b	This column defines whether or not the association was observed in the other tissue type, and if so the uncorrected p value is given.  				
c	Identifiers can be linked to genomic regions at  https://www.affymetrix.com/site/login/login.affx						
d	Cross hybridization scoring system: 1=unique, 2= some overlap, 3= complete sequence identity elsewhere in the genome					
