# Supplementary material for: Tissue-Specific Genetic Control of Splicing: Implications for the Study of Complex Traits
Source: PLoS Biol. 2008 Dec 23;6(12):e1000001. doi: 10.1371/journal.pbio.1000001 (PMC2605930; doi:10.1371/journal.pbio.1000001)
Supplement: Table S4 — (285 KB RTF) [file pbio.1000001.st004.rtf]

Table S4.  Table of associations between consensus site SNPs and adjacent exons	
							
Consensus site SNP	Gene	Chr	Tissue affected	p valuea	Other tissue affected (Y/N)a		
rs10814567	POLR1E	9	BRAIN	2.24E-11	Y		
rs2251182	INADL	1	BRAIN	4.23E-07	Y		
rs12544854	ASAH1	8	BRAIN	6.38E-06	Y		
rs2297988	RRP12	10	BRAIN	4.62E-05	Y		
rs2424928	DNMT3B	20	BRAIN	1.98E-04	N		
rs2296788	USP20	9	BRAIN	6.96E-04	N		
rs1805377	XRCC4	5	BRAIN	7.33E-04	N		
rs2293028	RYR3	15	BRAIN	0.001	N		
rs2241573	PRDM10	11	BRAIN	0.002	N		
rs10278590	RARRES2	7	BRAIN	0.003	Y		
rs610382	JAM3	11	BRAIN	0.004	Y		
rs7999630	UPF3A	13	BRAIN	0.004	Y		
rs3735108	IQCE	7	BRAIN	0.004	Y		
rs2290771	WBP2	17	BRAIN	0.005	Y		
rs13028613	PLEKHH2	2	BRAIN	0.005	Y		
rs11083846	PRKD2	19	BRAIN	0.006	N		
rs10897526	MAP4K2	11	BRAIN	0.006	Y		
rs3736242	SMAD7	18	BRAIN	0.007	N		
rs2360712	DOCK8	9	BRAIN	0.007	N		
rs10271133	CUL1	7	BRAIN	0.007	N		
rs6577877	FAM135B	8	BRAIN	0.008	N		
rs12202302	RPS12	6	BRAIN	0.009	N		
rs2073711	CILP	15	BRAIN	0.01	N		
rs26279	MSH3	5	BRAIN	0.01	N		
rs521040	CLEC1B 	12	BRAIN	0.012	N		
rs1465131	NEDD9	6	BRAIN	0.013	N		
rs10260691	NUP205	7	BRAIN	0.013	N		
rs3824700	MYO3A	10	BRAIN	0.014	N		
rs4937387	C11orf45	11	BRAIN	0.014	N		
rs2455834	ANKRD28	3	BRAIN	0.015	Y		
rs3762636	KIF15	3	BRAIN	0.017	N		
rs2243831	KIAA0319	6	BRAIN	0.018	N		
rs2125739	ABCC10	6	BRAIN	0.018	Y		
rs2769154	PALM2	9	BRAIN	0.019	N		
rs2074697	SSPO	7	BRAIN	0.02	N		
rs3768434	CDC42BPA	1	BRAIN	0.02	N		
rs2156634	GRIK4	11	BRAIN	0.02	N		
rs4627097	EI24	11	BRAIN	0.021	N		
rs225358	TFF1	21	BRAIN	0.021	N		
rs4842254	C9orf62	9	BRAIN	0.023	N		
rs747250	FLJ34521	11	BRAIN	0.023	N		
rs501168	TJP3	19	BRAIN	0.024	N		
rs2290251	EVPL	17	BRAIN	0.024	N		
rs11046589	MFAP5	12	BRAIN	0.025	N		
rs2272720	MYOM2	8	BRAIN	0.026	N		
rs12914825	RYR3	15	BRAIN	0.026	N		
rs3742261	DGKH	13	BRAIN	0.026	N		
rs16970025	B4GALNT2	17	BRAIN	0.027	N		
rs2286343	EVC	4	BRAIN	0.03	N		
rs12148472	CTSH	15	BRAIN	0.033	Y		
rs7851787	COL15A1	9	BRAIN	0.034	N		
rs4889294	BCMO1	16	BRAIN	0.037	N		
rs1333234	GLT6D1	9	BRAIN	0.038	N		
rs12562197	---	1	BRAIN	0.041	N		
rs4698795	RRH	4	BRAIN	0.045	N		
rs2284923	RNF8	6	BRAIN	0.046	N		
rs646406	FBXO18	10	BRAIN	0.046	N		
rs710865	---	1	BRAIN	0.047	N		
rs3821831	ITIH4	3	BRAIN	0.047	N		
rs8110220	HSD17B14	19	BRAIN	0.048	N		
rs1998721	CACNA1S	1	BRAIN	0.049	N		
rs140524	NCAPH2	22	BRAIN	0.049	N		
rs7770794	PIP3-E	6	PBMC	1.23E-11	N		
rs739924	SPATA20	17	PBMC	1.24E-08	Y		
rs3762374	TMEM77	1	PBMC	2.65E-08	Y		
rs2863095	MRPL43	10	PBMC	4.66E-06	Y		
rs2289001	DENND3	8	PBMC	4.93E-05	N		
rs4641276	ZNF362	1	PBMC	1.37E-04	Y		
rs1827293	NBPF3	1	PBMC	1.91E-04	N		
rs206111	LOC646799	13	PBMC	7.14E-04	N		
rs790055	F11R	1	PBMC	0.001	N		
rs3811035	FCRL5	1	PBMC	0.001	N		
rs1466018	LOC654433	2	PBMC	0.001	Y		
rs2289564	NAV2	11	PBMC	0.001	N		
rs3737374	KIAA1012	18	PBMC	0.002	N		
rs6579767	KIAA0194	5	PBMC	0.004	N		
rs593690	PIWIL4	11	PBMC	0.004	N		
rs3751979	SPECC1	17	PBMC	0.004	N		
rs2274756	MMP9	20	PBMC	0.004	N		
rs2074071	---	19	PBMC	0.007	Y		
rs7104577	GDPD4	11	PBMC	0.007	Y		
rs3895736	TMEM89	3	PBMC	0.008	N		
rs654093	ALKBH8	11	PBMC	0.008	N		
rs2277984	C3	19	PBMC	0.009	N		
rs10982110	COL27A1	9	PBMC	0.009	N		
rs6494466	---	15	PBMC	0.01	N		
rs798949	C7orf58	7	PBMC	0.01	N		
rs745975	HNF4A	20	PBMC	0.01	N		
rs7774697	UHRF1BP1	6	PBMC	0.011	N		
rs3736591	LOC389541	7	PBMC	0.012	N		
rs1126159	LOC400696	19	PBMC	0.014	N		
rs726176	SORBS1	10	PBMC	0.014	N		
rs7199993	ZC3H7A	16	PBMC	0.015	N		
rs243834	MMP2	16	PBMC	0.019	N		
rs10927386	EFCAB2	1	PBMC	0.019	Y		
rs4142087	---	6	PBMC	0.02	N		
rs2281860	PDCD11	10	PBMC	0.022	N		
rs11712748	ZBTB11	3	PBMC	0.024	N		
rs11633399	ZNF609	15	PBMC	0.026	N		
rs8100379	LOC388503	19	PBMC	0.026	N		
rs9863860	---	3	PBMC	0.029	Y		
rs11580603	PTPN14	1	PBMC	0.03	N		
rs3087409	WRN	8	PBMC	0.032	N		
rs10490182	DNER	2	PBMC	0.032	N		
rs6483642	NAV2	11	PBMC	0.033	N		
rs3212310	EZR	6	PBMC	0.035	N		
rs1519654	NMS	2	PBMC	0.037	N		
rs16926628	MYO3A	10	PBMC	0.037	N		
rs1264894	OVGP1	1	PBMC	0.038	N		
rs1551066	KCNAB1	3	PBMC	0.04	Y		
rs4733994	ADAM32	8	PBMC	0.04	N		
rs12823621	FGD4	12	PBMC	0.041	N		
rs7586144	CYBRD1	2	PBMC	0.044	N		
rs743810	TOM1	22	PBMC	0.046	N		
a  p values reflect lowest association observed for a SNP (uncorrected for multiple testing) 	
and a probeset quantifying an exon in a tissue type (other tissue type was significant with a 	
p value at or greater that reported on the table, but less than 0.05).  		
							
