# Supplementary material for: Tissue-Specific Genetic Control of Splicing: Implications for the Study of Complex Traits
Source: PLoS Biol. 2008 Dec 23;6(12):e1000001. doi: 10.1371/journal.pbio.1000001 (PMC2605930; doi:10.1371/journal.pbio.1000001)
Supplement: Table S5 — Full references are provided below this table. (459 KB RTF) [file pbio.1000001.st005.rtf]

Table S5.  A list of genome-wide association studies interrogated for significant associations that affect expression and splicing in the SNPExpress database.  	
Full references are provided below this table.								
Trait	Variant	Gene/region	P value	Paper	Expression changes in SNPExpress Database					
abdominal aortic aneurysm	rs10757278	CDKN2A/B	1.2 X 10-12	Helgadottir et al, 2008	N					
ALS	rs10260404	DPP6	5.0 X 10-8	Van es et al, 2008	N					
alzheimer's	rs4420638	APOE	5.3 X 10-34	Coon, et al 2007	N					
AMD	rs9332739	C2	1.0 X 10-12	Swaroop et al, 2007	N					
AMD	rs1061170	CFH	< 10-100	Swaroop et al, 2007	N					
AMD	rs10490924	LOC387715	< 10-100	Swaroop et al, 2007	N					
AMD	rs547154	C2	6.8 X 10-10	Swaroop et al, 2007	N					
AMD	rs11200638	HTRA1	1.3 X 10-10	Gibbs et al, 2008	N					
ankylosing spondylitis	rs11209032	IL23R	7.5 X 10-9	Burton et al, 2007	N					
ankylosing spondylitis	rs30187	ARTS1	3.4 X 10-10	Burton et al, 2007	N					
asthma	rs7216389	17q21	9 X 10-11	Moffat et al, 2007	N					
atrial fibrillation	rs2200733	PITX2	3.3 X 10-41	Gudjbartsson et al, 2007	N					
atrial fibrillation	rs10033464	PITX2	6.9 X 10-11	Gudjbartsson et al, 2007	N					
bipolar	rs420259	16p12	6.3 X 10-8	WTCCC, 2007	N					
cancer	rs6983267	8q24	1.3 X 10-14	Tomlinson et al, 2007	N					
cancer	rs1859962	17q24.3	2.5 X 10-10	Gudmundsson et al, 2007b	N					
cancer	rs4430796	TCF2	1.4 X 10-11	Gudmundsson et al, 2007b	N					
cancer	rs1447295	8q24	4.0 X 10-15	Gudmundsson et al, 2007a	N					
cancer	rs16901979	8q24	1.0 X 10-9	Gudmundsson et al, 2007a	N					
cancer	rs10993994	MSMB	8.7 X 10-29	Eeles et al, 2008	N					
cancer	rs9364554	SLC22A3	5.5 X 10- 10	Eeles et al, 2008	N					
cancer	rs2660753	3p11	2.7 X 10-8	Eeles et al, 2008	N					
cancer	rs7931342	11q13	1.7 X 10-12	Eeles et al, 2008	N					
cancer	rs2735839	KLK3	1.5 X 10-18	Eeles et al, 2008	N					
cancer	rs5945619	NUDT11	1.5 X 10- 9	Eeles et al, 2008	N					
cancer	rs6465657	LMTK2	1.1 X 10-9	Eeles et al, 2008	N					
cancer	rs889312	MAP3K1	7 X 10-20	Easton et al, 2007	N					
cancer	rs3817198	LSP1	3 X 10-9	Easton et al, 2007	N					
cancer	rs2981582	FGFR2	2 X 10-76	Easton et al, 2007	N					
cancer	rs3803662	TNRC9/LOC643714	1 X 10-36	Easton et al, 2007	N					
cancer	rs12443621	TNRC9/LOC643714	2 X 10-19	Easton et al, 2007	N					
cancer	rs13281615	8q24	5 X 10-12	Easton et al, 2007	N					
cancer	rs8051542	TNRC9/LOC643714	1 X 10-12	Easton et al, 2007	N					
cancer	rs4939827	SMAD7	1.0  X 10-12	Broderick et al, 2007	N					
celiac disease	rs6822844	IL21	1.3 X 10-14	Van heel et al, 2007	N					
celiac disease	rs2187668	HLA-DQA1	< 10-19	Van heel et al, 2007	N					
celiac disease	rs9275141	HLA-DQA1/B1	3.9 X 10-16	Van heel et al, 2007	N					
celiac disease	rs9357152	HLA-DQA1/B1	5.2 X 10-14	Van heel et al, 2007	N					
coronary artery disease	rs10757278	CDKN2A/B	1.2 X 10-23	Helgadottir et al, 2008	N					
Crohn's	rs10210302	ATG16L1	7.1 X 10-14	WTCCC, 2007	N					
Crohn's	rs9858542	3p21	3.58 X 10-8	WTCCC, 2007	Y					
Crohn's	rs17234657	5p13.1	2.1 X 10-13	WTCCC, 2007	N					
Crohn's	rs11805303	IL23R	6.5 X 10-13	WTCCC, 2007	N					
Crohn's	rs2076756	CARD15	7.0 X 10-14	Rioux, et al, 2007	N					
Crohn's	rs7517847	IL23R	3.0 X 10-12	Rioux, et al, 2007	N					
Crohn's	rs2414476	15q21.3	8.71 X 10-9	Raelson et al, 2007	N					
Crohn's	rs11208994	1p31.3	1.51 X 10-8	Raelson et al, 2007	N					
Crohn's	rs2542151	PTPN2	3.2 X 10-8	Parkes et al, 2007	N					
Crohn's	rs10883365	NKX2-3	3.7 X 10-10	Parkes et al, 2007	N					
Crohn's	rs10077785	IBD5	7.5 X 10-8	Parkes et al, 2007	N					
Crohn's	rs13361189	IRGM	2.1 X 10-10	Parkes et al, 2007	N					
Crohn's	rs11209026	IL23R	2.2 X 10-18	libioulle et al, 2007	N					
HDL cholesterol	rs2144300	GALNT2	2.6 X 10-14	Willer et al, 2008	N					
HDL cholesterol	rs2156552	LIPG	6.4 X 10-12	Willer et al, 2008	N					
HDL cholesterol	rs2338104	MVK/MMAB	3.4 X 10-8	Willer et al, 2008	N					
HDL cholesterol	rs3764261	CETP	2.3 X 10-57	Willer et al, 2008	N					
HDL cholesterol	rs4149274	ABCA1	7.4 X 10-8	Willer et al, 2008	N					
HDL cholesterol	rs4775041	LIPC	3.2 X 10-20	Willer et al, 2008	N					
HDL cholesterol	rs1800775	CETP	1. X 10-73	Kathiresan et al, 2008	N					
HDL cholesterol	rs1800588	LIPC	2 X 10-32	Kathiresan et al, 2008	N					
HDL cholesterol	rs328	LPL	9 X 10-23	Kathiresan et al, 2008	N					
HDL cholesterol	rs3890182	ABCA1	3 X 10-10	Kathiresan et al, 2008	N					
height	rs1042725	HMGA2	4 X 10-16	Weedon et al, 2007	N					
HIV-viral load at setpoint	rs2395029	HCP5	9.36 X 10-12	Fellay et al, 2007	N					
HIV-viral load at setpoint	rs9264942	HLA-C	3.77 X 10-9	Fellay et al, 2007	N					
LDL cholesterol	rs599839	1p13	6.1 X 10-33	Willer et al, 2008	N					
LDL cholesterol	rs16996148	19p13	2.5 X 10-9	Willer et al, 2008	N					
LDL cholesterol	rs17145738	MLXIPL	2.0 X 10-12	Willer et al, 2008	N					
LDL cholesterol	rs1748195	ANGPTL3	1.7 X 10-10	Willer et al, 2008	N					
LDL cholesterol	rs4775041	LIPC	1.6 X 10-8	Willer et al, 2008	N					
LDL cholesterol	rs562338	APOB	5.6 X 10-22	Willer et al, 2008	N					
LDL cholesterol	rs6511720	LDLR	4.2 X 10-26	Willer et al, 2008	N					
LDL cholesterol	rs4420638	APOE/APOC cluster	1 X 10-60	Kathiresan et al, 2008	N					
LDL cholesterol	rs11591147	PCSK9	2. X 10-44	Kathiresan et al, 2008	N					
LDL cholesterol	rs12654264	HMGCR	1 X 10-20	Kathiresan et al, 2008	N					
LDL cholesterol	rs599838	1p13	5 X 10-42	Kathiresan et al, 2008	N					
lupus	rs7574865	STAT4	9 X 10-14	Hom et al, 2008	N					
lupus	rs13277113	C8orf13/BLK	1.1 X 10- 10	Hom et al, 2008	N					
lupus	rs2187668	HLA-DRB1*0301	3 X 10-21	Hom et al, 2008	N					
lupus	rs10488631	IRF5/TNPO3	2 X 10-11	Hom et al, 2008	N					
lupus	rs11574637	ITGAM–ITGAX	3 X 10-11	Hom et al, 2008	N					
lupus	rs5754217	UBE2L3	7.5 X 1008	Harley et al, 2008	N					
lupus	rs6445975	PXK	7.1 X 10-9	Harley et al, 2008	N					
lupus	rs7829816	LYN	5.4 X 10- 9	Harley et al, 2008	N					
lupus	rs12537284	IRF5/TNPO3	3.6 X 10-19	Harley et al, 2008	N					
lupus	rs4963128	KIAA1542	3.0 X 10- 10	Harley et al, 2008	N					
lupus	rs9275572	HLA	2.8 X 10-12	Harley et al, 2008	Y					
lupus	rs6985109	XKR6	2.5 X 10-11	Harley et al, 2008	N					
lupus	rs6568431	6q21	1.7 X 10-8	Harley et al, 2008	N					
lupus	rs10798269	1q25.1	1.7 X 10-52	Harley et al, 2008	N					
lupus	rs9888739	ITGAM	1.6 X 10-23	Harley et al, 2008	N					
lupus	rs2431697	5q33	1.0 X 10-10	Harley et al, 2008	N					
lupus	rs10239340	IRF5/TNPO3	7.0 X 10-16	Harley et al, 2008	Y					
lupus	rs2248932	BLK	7.0 X 10-10	Harley et al, 2008	N					
lupus	rs7836059	C8orf12	4.0 X 10-10	Harley et al, 2008	N					
MS	rs12722489	IL2RA	2.96 X 10-8	Hafler et al, 2007	N					
MS	rs3135388	HLA-DRA	8.94 X 10-81	Hafler et al, 2007	N					
obesity	rs9930506	FTO	3.4 X 10-8	Scuteri et al, 2007	N					
pigment	rs12821256	KITLG	3.8 X 10-30	Sulem et al, 2007	N					
pigment	rs1540771	6p25.3b	3.7 X 10-18	Sulem et al, 2007	N					
pigment	rs1393350	TYR	3.3 X 10-12	Sulem et al, 2007	N					
pigment	rs1805007	MC1R	2.0 X 10-142	Sulem et al, 2007	N					
pigment	rs12896399	SLC24A4	1.4 X 10-48	Sulem et al, 2007	N					
pigment	rs1667394	OCA2	1.3 X 10-241	Sulem et al, 2007	N					
pigment	rs1042602	TYR	1.5 X 10-11	Sulem et al, 2007	N					
pigment	rs1805008	MC1R	4.2 X 10-95	Sulem et al, 2007	N					
pigment	rs7495174	OCA2	3.0 X 10-24	Sulem et al, 2007	N					
QT interval	rs10494366	NOS1AP	<10-10	Arking et al, 2006	N					
restless leg syndrome	rs1026732	MAP2K5	1.44 X 10-11	Winkelmann et al, 2007	N					
restless leg syndrome	rs2300478	MEIS1	8.1 X 10-23	Winkelmann et al, 2007	N					
restless leg syndrome	rs9296249	BTBD9	9.4 X 10-13	Winkelmann et al, 2007	N					
restless leg syndrome	rs3923809	BTBD9	3 X 10-14	Stefansson et al, 2007	N					
rheumatoid arthritis	rs615672	HLA-DRB1	7.5 X 10-27	WTCCC, 2007	N					
rheumatoid arthritis	rs6457617	MHC	5.18 X 10-75	WTCCC, 2007	Y					
rheumatoid arthritis	rs6920220	6q23	3.6 X 10-12	Thomson et al, 2007	N					
rheumatoid arthritis	rs3761847	TRAF1-C5	4 X 10-14	Plenge et al, 2007b	N					
rheumatoid arthritis	rs10499194	TNFAIP3/OLIG3	1 X 10-9	Plenge et al, 2007a	N					
rheumatoid arthritis	rs6679677	PTPN22	5.55 X 10-25	WTCCC, 2007	Y					
schizophrenia	rs4129148	CSF2RA	3.7 X 10-7	Lencz et al, 2007	N					
serum urate	rs7442295	SLC2A9	2 X 10-15	Wallace et al, 2008	N					
triglycerides	rs780094	GCKR	6.1 X 10-32	Willer et al, 2008	N					
triglycerides	rs12286037	APOA5	1.0 X 10-26	Willer et al, 2008	N					
triglycerides	rs6589566	APOA5	2.9 X 10-11	Wallace et al, 2008	N					
triglycerides	rs12130333	1p31	2 X 10-8	Kathiresan et al, 2008	N					
triglycerides	rs16996148	19p13	4 X 10-9	Kathiresan et al, 2008	N					
triglycerides	rs17145738	MLXIPL	7 X 10-22	Kathiresan et al, 2008	N					
triglycerides	rs17321515	TRIB1	4 X 10-17	Kathiresan et al, 2008	N					
triglycerides	rs328	LPL	2 X 10-28	Kathiresan et al, 2008	N					
triglycerides	rs4846914	GALNT2	7 X 10-15	Kathiresan et al, 2008	N					
type 1 diabetes	rs9272346	MHC	5.5 X 10-134	WTCCC, 2007	N					
type 1 diabetes	rs3764021	12p13	5.1 X 10-8	WTCCC, 2007	N					
type 1 diabetes	rs9270986	HLA-DRB1	2.3 X 10-122	WTCCC, 2007	Y					
type 1 diabetes	rs2542151	PTPN2	1.2 X 10-14	Todd et al, 2007	N					
type 1 diabetes	rs17696736	C12orf30	2.3 X 10-16	Todd et al, 2007	N					
type 1 diabetes	rs12708716	KIAA0350	2.6 X 10-18	Todd et al, 2007	N					
type 1 diabetes	rs1990760	IFIH1	1.8 X 10-11	Todd et al, 2007	N					
type 1 diabetes	rs2292239	ERBB3	1.5 X 10-20	Todd et al, 2007	N					
type 1 diabetes	rs6679677	PTPN22	2.1 X 10-80	Todd et al, 2007	Y					
type 1 diabetes	rs763361	CD226	1.4 X 10-8	Todd et al, 2007	N					
type 1 diabetes	rs11171739	12q13	9.7 x 10^-11^	WTCCC, 2007	Y					
type 2 diabetes	rs1801282	PPARG	1.7 X 10-6	Scott et al, 2007	N					
type 2 diabetes	rs4402960	IGF2BP2	8.9 X 10-16	Diabetes genetics initiative, 2007	N					
type 2 diabetes	rs10811661	CDKN2A/B	7.8 X 10-15	Diabetes genetics initiative, 2007	N					
type 2 diabetes	rs5219	KCNJ11	6.7 X 10-11	Diabetes genetics initiative, 2007	N					
type 2 diabetes	rs1111875	HHEX	5.7 X 10-10	Diabetes genetics initiative, 2007	N					
type 2 diabetes	rs13266634	SLC30A8	5.3 X 10-8	Diabetes genetics initiative, 2007	N					
type 2 diabetes	rs7754840	CDKAL1	4.1 X 10-11	Diabetes genetics initiative, 2007	N					
type 2 diabetes	rs7903146	TCF7L2	1.0 X 10-48	Diabetes genetics initiative, 2007	N					
XFG (exfoliation glaucoma)	rs3825942	LOXL1	3.0 X 10-21	Thorleifsson et al, 2007	N					
XFG (exfoliation glaucoma)	rs1048661	LOXL1	2.3 X 10-12	Thorleifsson et al, 2007	N					

Supplemental references
1.	Arking DE, Pfeufer A, Post W, Kao WH, Newton-Cheh C, et al. (2006) A common genetic variant in the NOS1 regulator NOS1AP modulates cardiac repolarization. Nat Genet 38: 644-651. Epub 2006 Apr 2030.
2.	Broderick P, Carvajal-Carmona L, Pittman AM, Webb E, Howarth K, et al. (2007) A genome-wide association study shows that common alleles of SMAD7 influence colorectal cancer risk. Nat Genet 39: 1315-1317. Epub 2007 Oct 1314.
3.	Burton PR, Clayton DG, Cardon LR, Craddock N, Deloukas P, et al. (2007) Association scan of 14,500 nonsynonymous SNPs in four diseases identifies autoimmunity variants. Nat Genet 39: 1329-1337.
4.	Consortium WTCC (2007) Genome-wide association study of 14,000 cases of seven common diseases and 3,000 shared controls.  447: 661-678.
5.	Coon KD, Myers AJ, Craig DW, Webster JA, Pearson JV, et al. (2007) A high-density whole-genome association study reveals that APOE is the major susceptibility gene for sporadic late-onset Alzheimer's disease. J Clin Psychiatry 68: 613-618.
6.	Diabetes Genetics Initiative of Broad Institute of Harvard and MIT LU, and Novartis Institutes of BioMedical Research,  , Saxena R, Voight BF, Lyssenko V, Burtt NP, et al. (2007) Genome-Wide Association Analysis Identifies Loci for Type 2 Diabetes and Triglyceride Levels. Science 316: 1331-1336.
7.	Easton DF, Pooley KA, Dunning AM, Pharoah PD, Thompson D, et al. (2007) Genome-wide association study identifies novel breast cancer susceptibility loci. Nature 447: 1087-1093.
8.	Eeles RA, Kote-Jarai Z, Giles GG, Olama AA, Guy M, et al. (2008) Multiple newly identified loci associated with prostate cancer susceptibility. Nat Genet.
9.	Fellay J, Shianna KV, Ge D, Colombo S, Ledergerber B, et al. (2007) A whole-genome association study of major determinants for host control of HIV-1. Science 317: 944-947. Epub 2007 Jul 2019.
10.	Gibbs D, Yang Z, Constantine R, Ma X, Camp NJ, et al. (2008) Further mapping of 10q26 supports strong association of HTRA1 polymorphisms with age-related macular degeneration. Vision Res 48: 685-689.
11.	Gudbjartsson DF, Arnar DO, Helgadottir A, Gretarsdottir S, Holm H, et al. (2007) Variants conferring risk of atrial fibrillation on chromosome 4q25.  448: 353-357.
12.	Gudmundsson J, Sulem P, Manolescu A, Amundadottir LT, Gudbjartsson D, et al. (2007) Genome-wide association study identifies a second prostate cancer susceptibility variant at 8q24. Nat Genet 39: 631-637.
13.	Gudmundsson J, Sulem P, Steinthorsdottir V, Bergthorsson JT, Thorleifsson G, et al. (2007) Two variants on chromosome 17 confer prostate cancer risk, and the one in TCF2 protects against type 2 diabetes.  39: 977-983.
14.	Hafler DA, Compston A, Sawcer S, Lander ES, Daly MJ, et al. (2007) Risk alleles for multiple sclerosis identified by a genomewide study. N Engl J Med 357: 851-862.
15.	Harley JB, Alarcon-Riquelme ME, Criswell LA, Jacob CO, Kimberly RP, et al. (2008) Genome-wide association scan in women with systemic lupus erythematosus identifies susceptibility variants in ITGAM, PXK, KIAA1542 and other loci. Nat Genet 40: 204-210.
16.	Helgadottir A, Thorleifsson G, Magnusson KP, Gretarsdottir S, Steinthorsdottir V, et al. (2008) The same sequence variant on 9p21 associates with myocardial infarction, abdominal aortic aneurysm and intracranial aneurysm. Nat Genet 40: 217-224.
17.	Hom G, Graham RR, Modrek B, Taylor KE, Ortmann W, et al. (2008) Association of Systemic Lupus Erythematosus with C8orf13-BLK and ITGAM-ITGAX. N Engl J Med.
18.	Kathiresan S, Melander O, Guiducci C, Surti A, Burtt NP, et al. (2008) Six new loci associated with blood low-density lipoprotein cholesterol, high-density lipoprotein cholesterol or triglycerides in humans. Nat Genet 40: 189-197.
19.	Lencz T, Morgan TV, Athanasiou M, Dain B, Reed CR, et al. (2007) Converging evidence for a pseudoautosomal cytokine receptor gene locus in schizophrenia. Mol Psychiatry 12: 572-580.
20.	Libioulle C (2007) Novel Crohn disease locus identified by genome-wide association maps to a gene desert on 5p13.1 and modulates expression of PTGER4.  3: e58.
21.	Moffatt MF, Kabesch M, Liang L, Dixon AL, Strachan D, et al. (2007) Genetic variants regulating ORMDL3 expression contribute to the risk of childhood asthma. Nature 448: 470-473. Epub 2007 Jul 2004.
22.	Parkes M, Barrett JC, Prescott NJ, Tremelling M, Anderson CA, et al. (2007) Sequence variants in the autophagy gene IRGM and multiple other replicating loci contribute to Crohn's disease susceptibility. Nat Genet 39: 830-832.
23.	Plenge RM, Cotsapas C, Davies L, Price AL, de Bakker PI, et al. (2007) Two independent alleles at 6q23 associated with risk of rheumatoid arthritis. Nat Genet 39: 1477-1482.
24.	Plenge RM, Seielstad M, Padyukov L, Lee AT, Remmers EF, et al. (2007) TRAF1-C5 as a Risk Locus for Rheumatoid Arthritis -- A Genomewide Study. N Engl J Med 357: 1199-1209.
25.	Raelson JV, Little RD, Ruether A, Fournier H, Paquin B, et al. (2007) Genome-wide association study for Crohn's disease in the Quebec Founder Population identifies multiple validated disease loci. Proc Natl Acad Sci U S A 104: 14747-14752.
26.	Rioux JD, Xavier RJ, Taylor KD, Silverberg MS, Goyette P, et al. (2007) Genome-wide association study identifies new susceptibility loci for Crohn disease and implicates autophagy in disease pathogenesis. Nat Genet 39: 596-604.
27.	Scott LJ, Mohlke KL, Bonnycastle LL, Willer CJ, Li Y, et al. (2007) A Genome-Wide Association Study of Type 2 Diabetes in Finns Detects Multiple Susceptibility Variants.  Science 316: 1341-1345.
28.	Scuteri A, Sanna S, Chen WM, Uda M, Albai G, et al. (2007) Genome-Wide Association Scan Shows Genetic Variants in the FTO Gene Are Associated with Obesity-Related Traits. PLoS Genet 3: e115.
29.	Stefansson H, Rye DB, Hicks A, Petursson H, Ingason A, et al. (2007) A genetic risk factor for periodic limb movements in sleep. N Engl J Med 357: 639-647.
30.	Sulem P, Gudbjartsson DF, Stacey SN, Helgason A, Rafnar T, et al. (2007) Genetic determinants of hair, eye and skin pigmentation in Europeans. Nat Genet 21: 21.
31.	Swaroop A, Branham KE, Chen W, Abecasis G (2007) Genetic susceptibility to age-related macular degeneration: a paradigm for dissecting complex disease traits. Hum Mol Genet 16 Spec No. 2: R174-182.
32.	Thomson W, Barton A, Ke X, Eyre S, Hinks A, et al. (2007) Rheumatoid arthritis association at 6q23. Nat Genet 39: 1431-1433.
33.	Thorleifsson G, Magnusson KP, Sulem P, Walters GB, Gudbjartsson DF, et al. (2007) Common sequence variants in the LOXL1 gene confer susceptibility to exfoliation glaucoma. Science 317: 1397-1400. Epub 2007 Aug 1399.
34.	Todd JA, Walker NM, Cooper JD, Smyth DJ, Downes K, et al. (2007) Robust associations of four new chromosome regions from genome-wide analyses of type 1 diabetes. Nat Genet 39: 857-864.
35.	Tomlinson I, Webb E, Carvajal-Carmona L, Broderick P, Kemp Z, et al. (2007) A genome-wide association scan of tag SNPs identifies a susceptibility variant for colorectal cancer at 8q24.21. Nat Genet 39: 984-988. Epub 2007 Jul 2008.
36.	van Es MA, van Vught PW, Blauw HM, Franke L, Saris CG, et al. (2008) Genetic variation in DPP6 is associated with susceptibility to amyotrophic lateral sclerosis. Nat Genet 40: 29-31.
37.	van Heel DA, Franke L, Hunt KA, Gwilliam R, Zhernakova A, et al. (2007) A genome-wide association study for celiac disease identifies risk variants in the region harboring IL2 and IL21. Nat Genet 39: 827-829. Epub 2007 Jun 2010.
38.	Wallace C, Newhouse SJ, Braund P, Zhang F, Tobin M, et al. (2008) Genome-wide association study identifies genes for biomarkers of cardiovascular disease: serum urate and dyslipidemia. Am J Hum Genet 82: 139-149.
39.	Weedon MN, Lettre G, Freathy RM, Lindgren CM, Voight BF, et al. (2007) A common variant of HMGA2 is associated with adult and childhood height in the general population. Nat Genet 39: 1245-1250.
40.	Willer CJ, Sanna S, Jackson AU, Scuteri A, Bonnycastle LL, et al. (2008) Newly identified loci that influence lipid concentrations and risk of coronary artery disease. Nat Genet 40: 161-169.
41.	Winkelmann J, Schormair B, Lichtner P, Ripke S, Xiong L, et al. (2007) Genome-wide association study of restless legs syndrome identifies common variants in three genomic regions. Nat Genet 39: 1000-1006. Epub 2007 Jul 1018.
